# Interrogation of an Enzyme Library Reveals the Catalytic Plasticity of Naturally Evolved [4+2] Cyclases

**DOI:** 10.1002/cbic.202300382

**Published:** 2023-06-18

**Authors:** Katja Zorn, Catherine R. Back, Rob Barringer, Veronika Chadimová, Monserrat Manzo‐Ruiz, Sbusisiwe Z. Mbatha, Juan‐Carlos Mobarec, Sam E. Williams, Marc W. van der Kamp, Paul R. Race, Christine L. Willis, Martin A. Hayes

**Affiliations:** ^1^ Compound Synthesis and Management, Discovery Sciences Biopharmaceuticals R&D AstraZeneca Pepparedsleden 1 431 83 Mölndal Sweden; ^2^ School of Biochemistry University of Bristol Bristol BS8 1TD UK; ^3^ School of Chemistry University of Bristol Bristol BS8 1TS UK; ^4^ Mechanistic and Structural Biology Biopharmaceuticals R&D AstraZeneca Cambridge CB21 6GH UK

**Keywords:** cyclases, biosynthetic gene cluster, polyketides, enzyme screening, intra-molecular Diels-Alder reaction

## Abstract

Stereoselective carbon‐carbon bond forming reactions are quintessential transformations in organic synthesis. One example is the Diels‐Alder reaction, a [4+2] cycloaddition between a conjugated diene and a dienophile to form cyclohexenes. The development of biocatalysts for this reaction is paramount for unlocking sustainable routes to a plethora of important molecules. To obtain a comprehensive understanding of naturally evolved [4+2] cyclases, and to identify hitherto uncharacterised biocatalysts for this reaction, we constructed a library comprising forty‐five enzymes with reported or predicted [4+2] cycloaddition activity. Thirty‐one library members were successfully produced in recombinant form. *In vitro* assays employing a synthetic substrate incorporating a diene and a dienophile revealed broad‐ranging cycloaddition activity amongst these polypeptides. The hypothetical protein Cyc15 was found to catalyse an intramolecular cycloaddition to generate a novel spirotetronate. The crystal structure of this enzyme, along with docking studies, establishes the basis for stereoselectivity in Cyc15, as compared to other spirotetronate cyclases.

## Graphical abstract

Diels‐Alderases perform an essential step in the biosynthesis of bioactive spirotetronates. To expand the understanding of such enzymes a cyclase library was created, which made it possible to identify a novel spirotetronate cyclase from a metagenome mining approach. Structural elucidation of both the enzyme by X‐ray crystallography and the product by NMR helped us to gain further insights into the essential features of how these enzymes perform complex cyclisations.

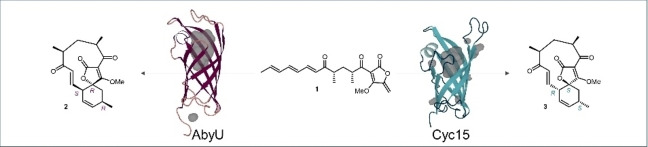

## Introduction

The Diels‐Alder reaction is widely employed in the synthesis of cyclohexenes, leveraging its utility in forming two new carbon‐carbon bonds and up to four new stereocentres in a single step.^[1]^ Although putative Diels‐Alder adducts have long been identified as potential intermediates in the biosynthesis of natural products, it is only recently that studies have enabled the formal identification and characterisation of the naturally evolved biocatalysts responsible for these key cyclisations.^[2]^ There are many challenges implicit in such studies, including the requirement to access often complex and unstable enzyme substrates (either by total synthesis or isolation from engineered microorganisms), the need to develop appropriate *in vitro* and *in vivo* assay conditions, and the detailed structural and computational studies that must be undertaken to gain mechanistic insights into the cyclases themselves.^[3]^ Over the past decade >100 putative [4+2] cyclases have been identified in a variety of biosynthetic gene clusters, highlighting the breadth of potential natural product scaffolds assembled using this transformation, along with the variety of protein folds which may facilitate [4+2] cycloadditions. Establishing a comprehensive understanding of naturally evolved [4+2] cyclases, specifically their catalytic mechanisms, molecular structures and substrate selectivities, could unlock the potential of these biocatalysts as tools in synthesis.

**Scheme 1:**
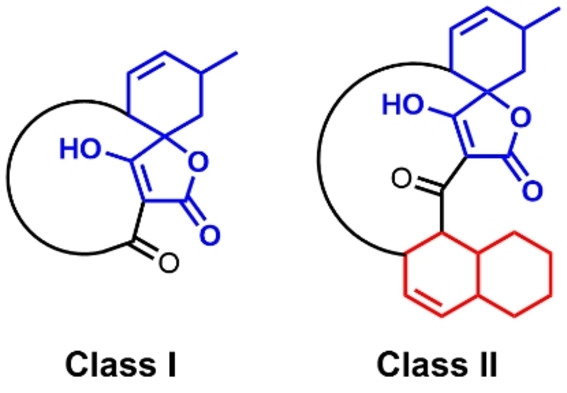
General structures of the two classes of spirotetronate polyketides.

Two well studied families of [4+2] cyclases are the spirotetronate cyclases and the decalin forming cyclases. These enzymes are both involved in the biosynthesis of the spirotetronate family of polyketide natural products.^[4]^ Members of this group of compounds exhibit potent bioactivities, including acting as antimicrobial, antiviral and/or anticancer agents. Spirotetronate natural products are broadly divided into distinct two classes (Scheme 1); class I compounds, which comprise a spirotetronate moiety embedded within a macrocycle of varying size, and class II compounds, which house an additional decalin ring system.[^2b^, ^5^] The biosynthesis of these molecules involves the action of a dedicated modular polyketide synthase (PKS), complimented by a suite of tailoring enzymes, which are orchestrated to assemble the final pathway product (Scheme S1).[^5a^, ^6^] Spirotetronate cyclases function in the formation of both class I and class II compounds, however, the decalin‐forming enzymes are unique to class II pathways. Interestingly, these two families of enzymes differ significantly in both their molecular architectures and catalytic mechanisms.[^5b^, ^7^] To date, five enzymes catalysing spirotetronate formation have been characterised in detail, PyrI4,^[7]^ AbyU,^[8]^ AbmU,^[9]^ AbnU^[10]^ and PloI4.^[11]^

Intriguingly, the spirotetronate cyclases exhibit modest primary amino acid sequence identity (<25 %; Tables S2 and S3), but retain a common overall fold. This comprises an eight‐stranded β‐barrel within which the barrel lumen constitutes the enzyme active site. Access to this site is regulated via a flexible capping loop, with the opposing barrel ‘head’ sealed by a salt bridge (Figure S1). Mechanistic studies indicate that these enzymes accelerate [4+2] cycloadditions via an asynchronous concerted mechanism, consistent with a formal Diels‐Alder reaction. Based on structural and mechanistic studies of spirotetronate cyclases conducted to date, there is no evidence to indicate the retention of specific structural features within the active sites of these biocatalysts that are implicitly required to facilitate catalysis. To improve our understanding of [4+2] cyclases and in particular those responsible for spirotetronate formation, here we report the construction and interrogation of a naturally evolved [4+2] cyclase library, screened using the synthesised *O*‐methylated tetronate substrate **1**. These studies identified a highly active [4+2] cyclase, whose deviant stereoselectivity was rationalised following X‐ray crystal structure elucidation in tandem with docking studies.

## Results and Discussion

### Generation of a [4+2] cyclase library

**Figure 1:**
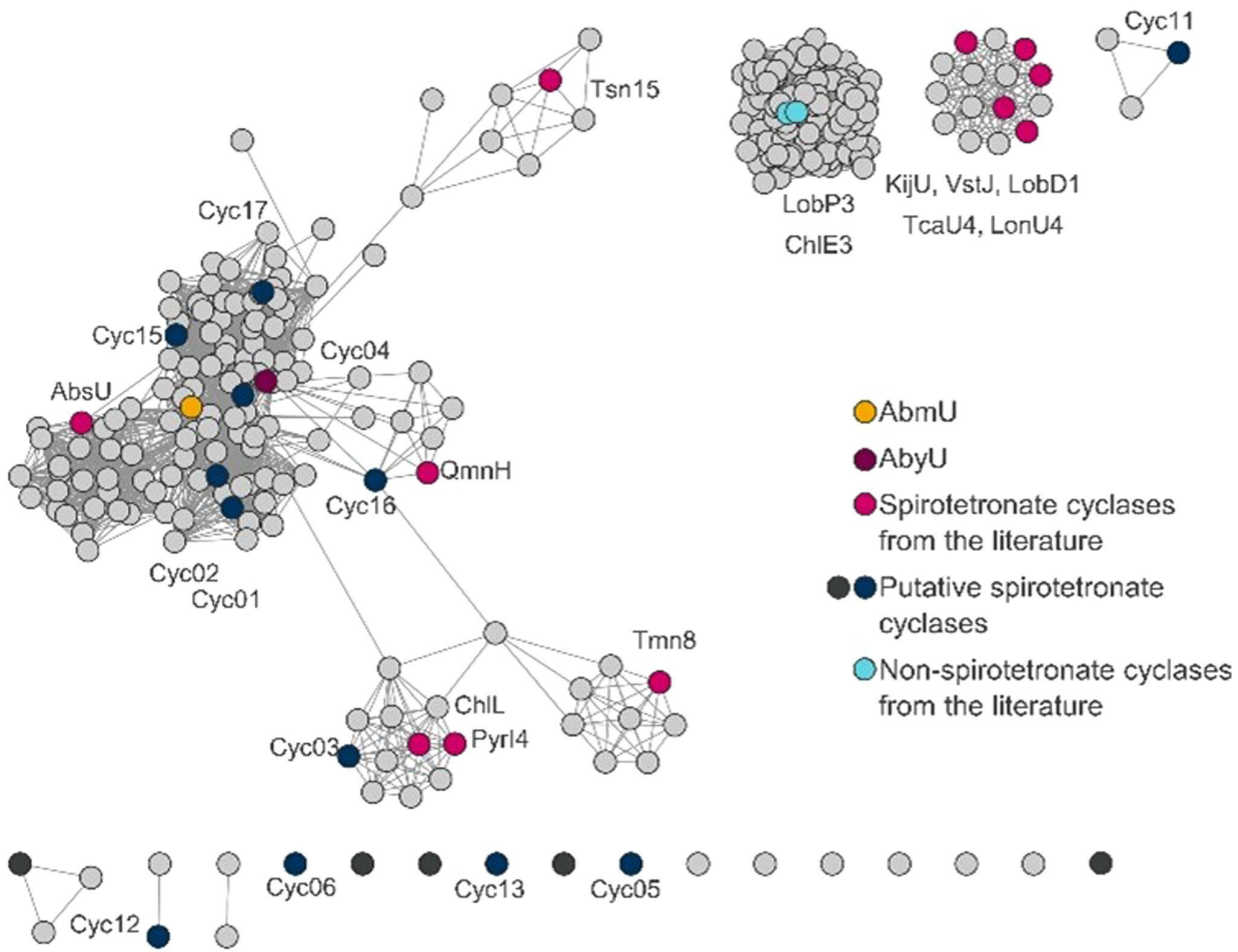
Sequence similarity network (SSN) based on spirotetronate cyclase sequences described in the literature. Displayed is the SSN based on 286 unique amino acid sequences with an alignment score of 15,^[15]^ AbmU (yellow), AbyU (magenta), and other spirotetronate cyclases reported in the literature (pink) are highlighted. 17 putative spirotetronate cyclase sequences (black & blue) were selected and after homology modelling studies 12 (blue) sequences were selected for further investigation.

As a starting point for library construction, sequences encoding 13 previously characterised spirotetronate cyclases were selected for inclusion (Table S2). These were complimented with the sequence of Tsn15, which possesses a characteristic spirotetronate cyclase eight‐stranded β‐barrel core fold, but which performs a pericyclic rearrangement of a six‐membered ring in tetronasin biosynthesis (Scheme S1B).^[6a]^ To expand the library yet further, 286 unique amino acid sequences were identified in public databases and assembled into a sequence similarity network (SSN, Figure 1). A cluster of 100 sequences was identified and excluded, since the 43 annotated sequences belonged to different protein‐fold families exhibiting cofactor binding motives like the two FAD‐dependent decalin forming cyclases ChlE3 and LobP3. Distinct clustering was observed for the known cyclases KijU, LobD1, LonU2, TcaU4, and VstJ, and for ChlL and PyrI4. From this SSN analysis 17 putative cyclase sequences were selected to cover different areas of the SSN (Table S4) and homology models generated for these polypeptides. Three methods were used: (i) RosettaCM^[12]^ as implemented in the Cyrus‐CAD bench application, (ii) a template based approach using YASARA,^[13]^ employing the reported crystal structures of AbmU, AbyU, PyrI4, Tsn15, and (iii) AlphaFold 2.0.^[14]^ Models which predicted eight stranded β‐barrel folds, consistent with those of known spirotetronate cyclases, were taken as an indication of the likely function of these enzymes. As a consequence of these analyses 12 of the 17 putative cyclase sequences were selected for inclusion in the library, with five candidates excluded.

With the release of AlphaFold 2.0 (AF2), it was possible to perform a full structural comparison of the 26 selected known and predicted cyclases (Figure S2). While AF2 could predict the core structure of the candidate cyclases with high confidence, in 60 % of the cases the terminal regions were not predicted to be structured. Furthermore, the candidate cyclases Cyc06, Cyc12, and Cyc13 were predicted to adopt non‐β‐barrel folds, with Cyc05 suggested to incorporate a large unstructured loop between strands β1 and β2. For the 23 structures which exhibit known or predicted β‐barrel folds, each houses a predominantly hydrophobic binding pocket within the barrel lumen, populated with a high proportion of aromatic residues. In many instances the enzyme binding pocket contains a pair of aromatic residues, which are resident on opposing sides of the barrel, whose side chains form π‐π stacking interactions, e. g. Trp124 and Phe41 in AbyU (Figure S1). Notably, such interactions are not observed in Tmn8 and Tsn15, which could account for the distinctive pericyclic rearrangements reported for these enzymes.[^6a^, ^16^] The predicted structures of these polypeptides are also distinct in that they each lack a salt‐bridge at the barrel ‘head’. In the majority of the 23 structures a glutamic acid and arginine pair fulfil this role, however, in PyrI4, ChlL, Cyc03, and Cyc04 the arginine residue is replaced by histidine. PyrI4, ChlL and Cyc03 reside in the same cluster in our SSN analysis and share an extended yet structured N‐terminal region, analogous to that reported in PyrI4.^[7]^ With the exception of Cyc05, each of our models possesses a 6–15 residue capping loop, which regulates access to the enzyme active site and may play a role in bringing the substrate into a reactive conformation.[^7^, ^8^]

Current models of catalysis in spirotetronate cyclases are derived predominantly from studies of AbmU,^[9]^ AbyU[^8a^, ^17^] and PyrI4.[^7^, ^18^] It is proposed that the substrate binding cavity is essential for forming an environment which brings the diene and dienophile into close proximity, thus facilitating the [4+2] cycloaddition reaction. There are no explicitly conserved active site residues shared by these three enzymes, and there is a general lack of mechanistic understanding with respect to the broader family of cyclases.[^3b^, ^3f^, ^19^] To establish if this plasticity impacts on substrate selectivity in the spirotetronate cyclases, we further expanded the scope of our cyclase library through inclusion of 19 additional candidate sequences from the literature (Table S5), to yield a 45‐member library.

We performed a phylogenetic analysis of the 45 cyclase library to explore the evolutionary relationships and potential functional differences among the cyclases (Figure S3). The spirotetronate‐forming cyclases clustered clearly together. The three abyssomicin forming Diels‐Alderases (AbmU, AbyU and AbsU) formed a clade with five putative cyclases, including Cyc17, which is likely involved in the production of a recently discovered class of spirotetronates the wychimicins.^[20]^ The phylogenetic analysis potentially explained the adoption of non‐β‐barrel folds in Cyc06, Cyc12, and Cyc13, with all three showing stronger evolutionary relationships with non‐spirotetranate forming cyclases.

To enable activity screening of library members, each enzyme was recombinantly over‐expressed in soluble form in *E. coli* and purified to homogeneity. To expedite this process we established a semi‐automated protein‐production workflow that enabled us to prepare 12 purified proteins per week and involved expression in auto‐induction media, followed by affinity and size exclusion chromatography. Protein identity and homogeneity were evaluated by SDS‐PAGE analysis, melting temperature (T_M_) studies and peptide mapping (Table S6). Of our target 45 proteins 31 were successfully produced using this workflow, including 12 spirotetronate cyclases previously described in the literature. The only exceptions were the individual N‐ and C‐terminal domains of QmnH which proved recalcitrant to production using this approach. Of the 12 selected sequences of putative spirotetronate cyclases, seven could be produced solubly and in high yields, with poor expression observed for Cyc01, Cyc02, Cyc06, Cyc16, and Cyc17 under our standard conditions. An additional 12 cyclases identified in the literature, but with no previously reported spirotetronate cyclase activity, were also solubly expressed and purified, while five cyclases from fungal sources (CcsF, EupF, gNR600, mAsR5, Sol5) and two bacterial enzymes (PyrE3, SpnF) were not expressed in sufficient quantities to enable further characterisation. Five proteins exhibited melting temperatures above 70 °C (AbyU, LonU2, PyrI4, Cyc03, and Cyc15). Although there was variation in purified protein yields, it was possible to obtain at least 1 mg of protein for 29 of the cyclases.

### Cyclase library screening using a synthetic substrate

**Figure 2:**
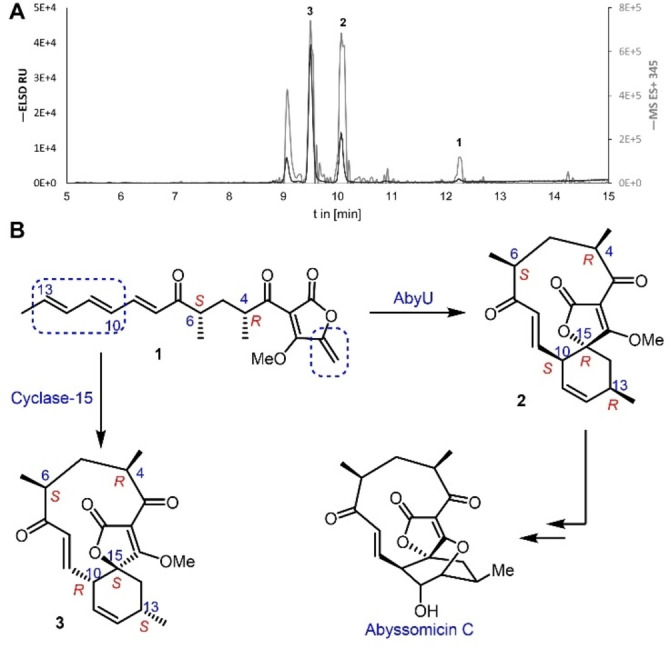
(**A**) Semi‐preparative HPLC trace of the scaled‐up Cyc15 assay with substrate **1** (t_R_=12.2 min) after 3 h incubation. Displayed are the ELSD (dark grey) and the extracted mass of the positive total ion current of the substrate and product ([M+H]+345 *m/z*; light grey) **1**. (**B**) Major products **2** and **3** isolated from *in vitro* assays with AbyU and Cyc15 respectively.

In an effort to explore the intramolecular cyclisation activities of library members a screening method was developed employing the *O*‐methylated substrate analogue **1** (Figure 2). Reactions were miniaturised to 5 μL so both the purified enzyme and tetronic acid derivative **1** could be valorised. Assays were conducted in 348‐well microtiter plates and comprised 1 mg/mL of protein in reaction buffer (20 mM Tris‐HCl, 150 mM NaCl, pH 7.5). Reactions were initiated by addition of 500 nL of the substrate stock solution (10 mM **1** in acetonitrile) using a nano‐scale liquid handler (Mosquito® HV) and were incubated for 20 min at 25 °C.

**Figure 3:**
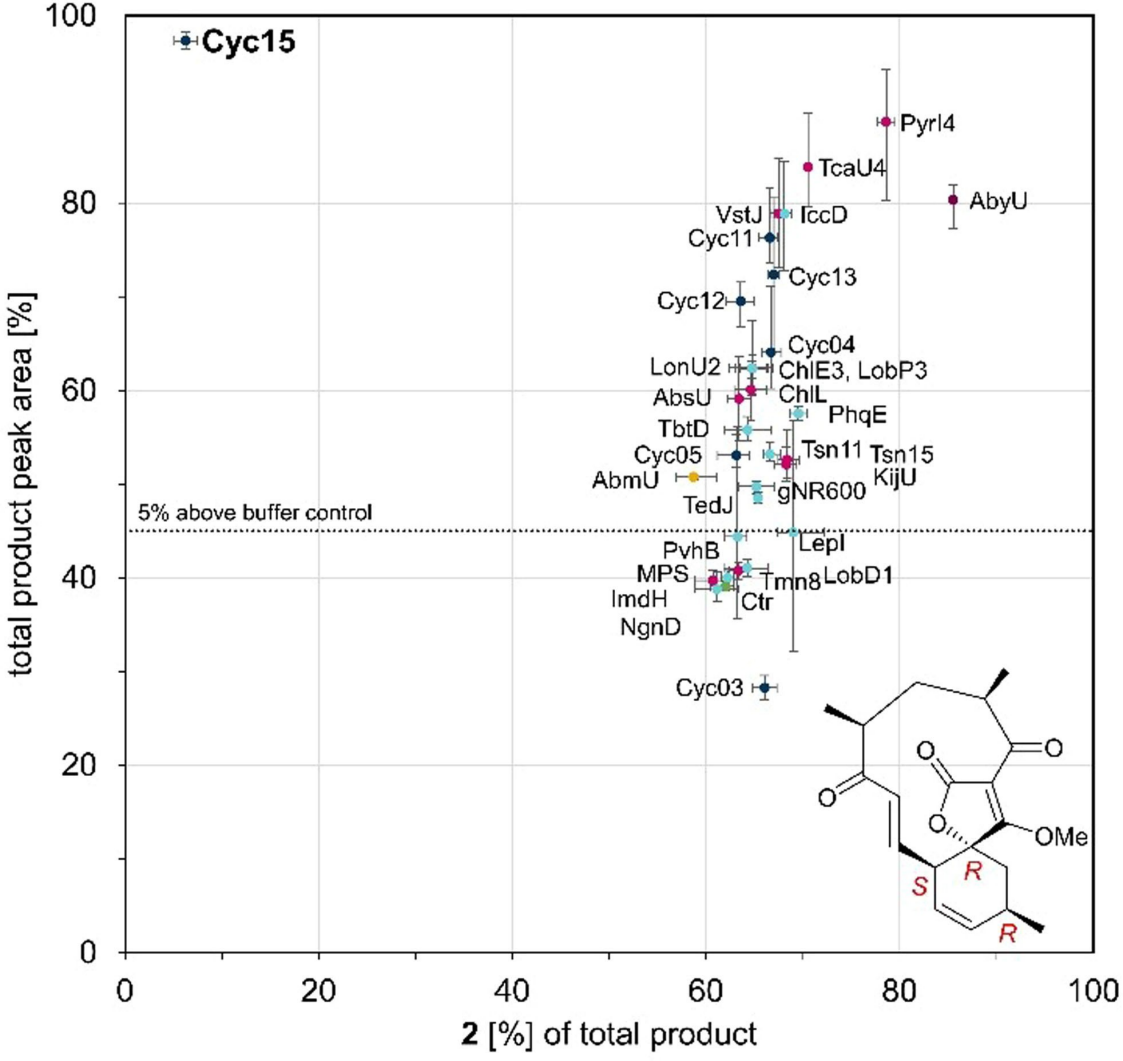
Analysis of product formation in biotransformations with cyclase library enzymes (1 mg/mL) or no enzyme and substrate (1 mM) by evaluating the total product formation compared to the formation of displayed diastereoisomer as percentage of the product fraction. Endpoints after incubations for 20 min with **1** were analysed by UPLC‐MS (QToF). The peak areas are resolved from the extracted mass of the total ion current of the substrate and the products (**1**‐**3**, [M+H]^+^ 345.170 *m/z*). Highlighting spirotetronate cyclases AbmU (yellow), AbyU (magenta), and others from literature (pink); putative spirotetronate cyclase (dark blue); non‐ spirotetronate cyclases (light blue); and the buffer control reaction (green, Ctr).

After quenching, high‐resolution UPLC‐MS (QToF) was used to analyse the reaction mixtures, with all assays performed in triplicate. Six reaction products were identified during library screening experiments, based on UPLC elution profile (retention times in the range 2.4–3.2 min), with substrate **1** eluting at 5.2 min (Figure S10). MS‐spectra of each peak confirmed the predicted mass of the expected protonated and sodium adduct of predicted cycloaddition products, [M+H]^+^ 345.170 *m/z* and [M+Na]^+^ 367.152 *m/z* (Figure S11). The principal product formed in the majority of reactions corresponded to **2**, the previously reported product of the AbyU catalysed transformation of **1**.[^8a^, ^17a^] More than 70 % of the tested cyclases showed a significant acceleration in the cyclisation of **1** (Figure 3). Most of the enzymes tested generated two or more products. Surprisingly, high overall conversion (>60 %) was observed for the decalin forming cyclases ChlE3, LobP3 and IccD with **1**. Due to the observed broad substrate selectivity of these enzymes it is presumed that their active site architectures are tolerant to chemically disparate substrates. Surprisingly both Cyc12 and Cyc13 gave products of the expected mass despite their AF2 models indicating folds distinct from that of a β‐barrel (Figure S2). Cyc15 showed the highest conversion of substrate **1** after 20 min whilst yielding the lowest quantity of product **2**. This enzyme forms four distinct products, including a predominant species with a retention time of 2.76 min (Figure S10). Cyc15 shares 28 % sequence identity with AbyU and was identified in the draft genome of *Streptomyces* sp. NL15‐2 K as a hypothetical protein (Table S4). Due to the fragmented nature of the biosynthetic gene cluster within which Cyc15 resides, it is not possible to assess the authentic function of this enzyme in *Streptomyces* sp. NL15‐2 K, or to infer the identity of the compound generated by the encoded biosynthetic pathway.

#### Structural elucidation of the major Cyc15 reaction product

To elucidate the structure of the reaction product of the Cyc15 catalysed transformation of **1**, this reaction was scaled‐up to 39 mL, employing equivalent reaction conditions to those used at 5 μL scale. The reaction was monitored by reverse‐phase HPLC‐MS and quenched after 3 h (Figure 2A). Whilst a minor amount of starting material **1** remained, new peaks were apparent including one which had the same retention time as spirotetronate **2** (t_R_=10.07 min, with the 10*S*, 13*R*, 15*R* configuration).^[8a]^ The major product (t_R_=9.51 min, 41 % peak area) was purified by semi‐preparative reverse‐phase HPLC and its structure was determined by extensive NMR studies.

It was evident that the new compound had the same connectivity as **2**. However, there were significant differences in their spectra (Figure S5‐9 and Table S1). For example, in the ^1^H‐NMR of AbyU product **2** in CDCl_3_, the signals assigned to 8‐H and 9‐H appeared at δ6.24 (d *J* 16.5 Hz) and δ6.46 (dd, *J* 16.5, 7.0 Hz) whereas in the Cyc15 product the signal assigned to 8‐H resonated at δ6.57 (d, *J* 16 Hz) and 9‐H at δ6.37 (dd *J* 16, 9.5 Hz). 2D‐NMR and ROESY studies confirmed the Cyc15 product to be the novel spirotetronate **3** with the (10*R*, 13*S*, 15*S*) configuration (Figure S9). Therefore, Cyc15 folds substrate **1** to produce **3** in a similar conformation as reported for AbmU in the production of abyssomicin 2.[^9^, ^17b^] Hence, we turned our attention to structural and computational studies of Cyc15 to verify this proposal.

#### Elucidation of Cyc15 X‐ray crystal structure

**Figure 4:**
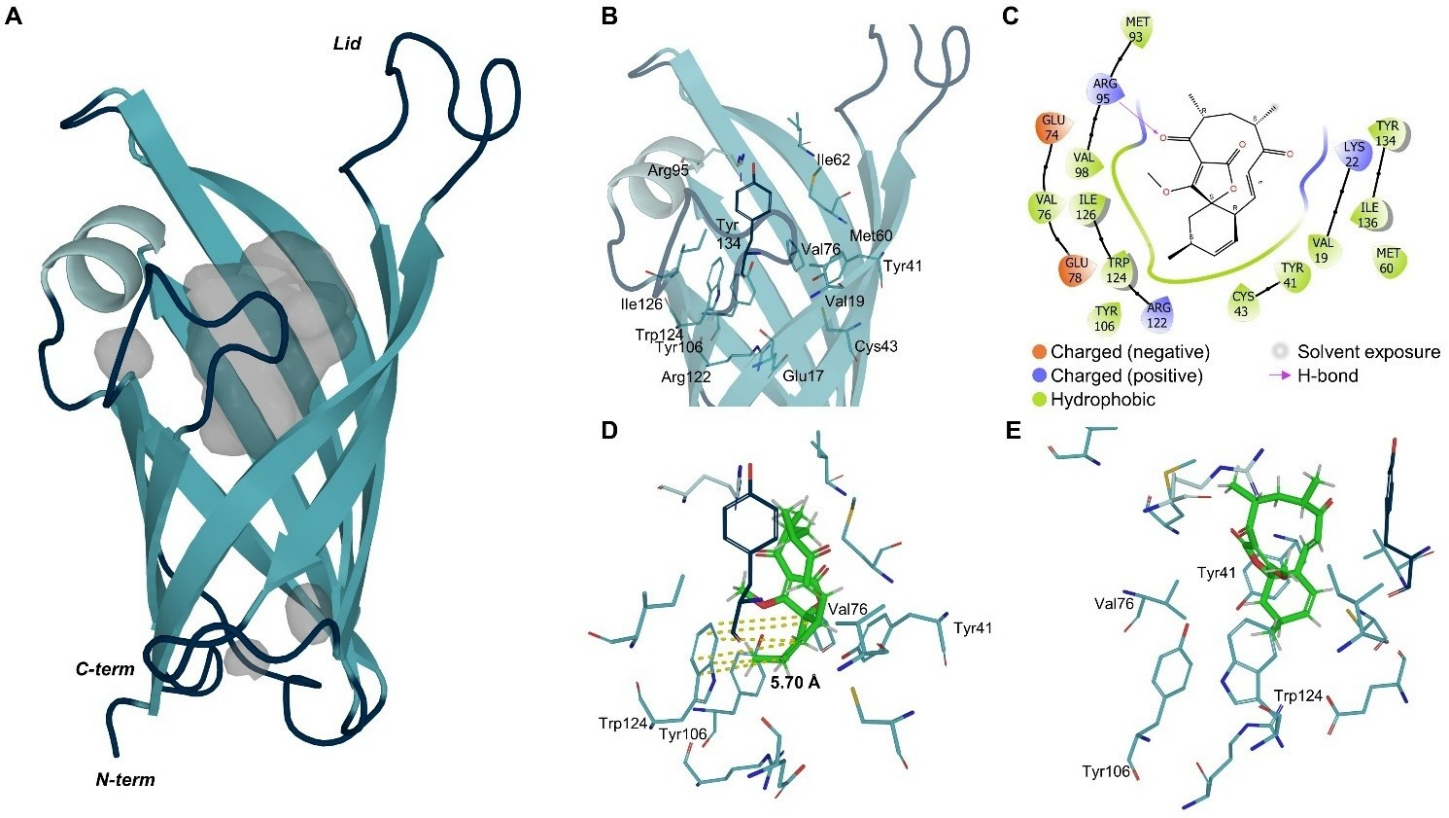
(**A**) Crystal structure of Cyc15 Chain A (8OF7) displayed as cartoon in blue with cavities in grey. (**B**) Residues of the active site are highlighted and labelled as sticks and (**C**) assigned from the docking studies to interact with product **3** binding. (**D**&**E**) Docked product **3** (green) from two perspectives is displayed in concert with the active site residues, highlighting the distance (yellow) of the cyclohexene ring towards Trp124.

To provide a structural basis for the Cyc15 catalysed reaction the crystal structure of this polypeptide was determined. The structure of Cyc15 (8OF7, Figure 4) was resolved to 1.7 Å using molecular replacement, employing an AlphaFold 2.0 generated search model of Cyc15. The structure reveals an asymmetric unit comprising two molecules of Cyc15 (Chain A and Chain B), which form a homodimer. Chain A comprises residues 9–148 of the full‐length polypeptide, with Chain B comprising residues 9–145. The overall fold of Cyc15 is analogous to those reported for other spirotetronate cyclases,[^7^, ^8^, ^9^] possessing an eight‐stranded antiparallel β‐barrel fold with (+1) 8 topology. The AlphaFold search model aligns well with our experimentally determined crystal structure with a Cα RMSD of 0.47 Å. The Cyc15 dimer interface is populated predominantly by hydrophobic residues, along with a pair of equivalently positioned cysteines (Cys59; one contributed by each monomer) which sit 5 Å apart. No evidence of disulfide bond formation is observed in our Cyc15 structure, however, this interaction may only become apparent under oxidising conditions. Each Cyc15 monomer houses a large, solvent exposed active site cavity, which is sealed at one end by a salt bridge formed between the residues Glu17 and Arg122. Access to this site is regulated via a flexible loop, formed by the β1‐β2 linker (residues Asn25‐Met36), which caps the opposing end of the barrel. In our Cyc15 crystal structure electron density is only observed for the capping loop of Chain A, which is packed tightly against a symmetry partner. As a consequence of this arrangement, the side chain of Glu132 from each copy of Chain A extends into the entrance of the central cavity of a symmetry partner, forcing the recipients capping loop into an ‘open’ conformation. The Cyc15 active site is populated by a combination of hydrophobic and aromatic amino acids consistent with the required local environment for promotion of the [4+2] cycloaddition (Figure 4).

#### Docking studies indicate the origin of stereoselectivity in Cyc15

It has been observed previously that docking of tetronic acid substrates into the active site of spirotetronate cyclases results in many possible binding modes.^[8a]^ As a consequence, we decided to investigate the stereoselectivity of Cyc15 using Glide^[21]^ docking studies with the two main diastereomeric products observed in this reaction (Figure 4C–E). The pose obtained with the (15*S*)‐configured product **3** has the correct configuration of the cyclohexene ring and aligns with an average distance of 5.7 Å towards the aromatic ring of Trp124. Binding of product **3** was favoured over product **2** indicated by lower binding energies (**2**: −4.50 kcal/mol; **3**: −5.45 kcal/mol, Figure S4). This underlines the experimental findings for Cyc15 selectivity and could indicate that formation of product **2** is partially due to auto‐cyclisation by the substrate in buffer. Furthermore, these docking results indicate that Arg95 may form a hydrogen bond with the carbonyl oxygen (C3=O), and there are also likely interactions with Lys22 and Tyr41. The macrocycle components interact with residues Val19, Lys22, Met60, Glu74, Met93, Arg95, and Tyr134; the tetronic acid group interacts with Val76, Met93, Val98, Tyr106, and Ile126; while the cyclohexene ring has interactions with Val19, Tyr41, Cys43, Val76, Glu78, Tyr106, Arg122, Trp124 and Ile136.

## Conclusion

In an effort to broaden our understanding of naturally evolved [4+2] cyclases, and to identify previously unreported biocatalysts for this reaction, we have generated a library of 45 known or putative cyclases, of which 31 have been produced in recombinant form. Screening of these enzymes using the synthetic tetronate substrate **1** revealed that an unexpectedly high proportion of these biocatalysts could transform this compound into one or more products. These data demonstrate that despite exhibiting modest amino acid sequence identity, these enzymes possess sufficient active site plasticity to enable the conversion of non‐cognate substrates to their respective minaturizcyclised products. A previously uncharacterised library member, Cyc15, which was identified using a genome mining approach, showed unprecedented catalytic activity with **1**, forming the novel cyclised product **3**. The structure of this compound was elucidated spectroscopically following the 8000‐fold scaling up of the Cyc15 reaction. **3** was found to be a diastereomer of **2** with the spiro‐centre in an (*S*)‐configuration. The X‐ray crystal structure of Cyc15 has been determined, and displays an eight‐stranded β‐barrel fold characteristic of the spirotetronate cyclases. Docking studies indicate a mode of substrate binding consistent with the formation of the diastereomer **3**, providing a structural explanation for the observed stereochemical output of the Cyc15 catalysed reaction. Our findings highlight the potential utility of naturally evolved [4+2] cyclases for the conversion of non‐cognate substrates and identify a novel spirotetronate cyclase with hitherto unreported stereoselectivity.

## Conflict of interest

The authors declare no conflict of interest.

1

## Supporting information

As a service to our authors and readers, this journal provides supporting information supplied by the authors. Such materials are peer reviewed and may be re‐organized for online delivery, but are not copy‐edited or typeset. Technical support issues arising from supporting information (other than missing files) should be addressed to the authors.

Supporting Information

## Data Availability

The data that support the findings of this study are available from the corresponding author upon reasonable request.
