# Supplementary material for: Interrogation of an Enzyme Library Reveals the Catalytic Plasticity of Naturally Evolved [4+2] Cyclases
Source: Chembiochem. 2023 Jun 18;24(14):e202300382. doi: 10.1002/cbic.202300382 (PMC10946715; doi:10.1002/cbic.202300382)
Supplement: Supplementary file 1 — Supporting Information [file CBIC-24-0-s001.pdf]

ChemBioChem

Supporting Information

Interrogation of an Enzyme Library Reveals the Catalytic Plasticity of Naturally Evolved [4+2] Cyclases

Katja Zorn, Catherine R. Back, Rob Barringer, Veronika Chadimová, Monserrat Manzo-Ruiz, Sbusisiwe Z. Mbatha, Juan-Carlos Mobarec, Sam E. Williams, Marc W. van der Kamp, Paul R. Race, Christine L. Willis, and Martin A. Hayes*

S1. Experimental Procedure

S1.1 Preparation of DNA constructs
S1.2 Sequence similarity network analysis
S1.3 Homology modelling
S1.4 Enzyme preparation
- S1.4.1 Protein expression
- S1.4.2 Protein purification with the streamlined protein supply process
- S1.4.3 Protein melting temperature determination
- S1.4.4 Protein concentration determinations
- S1.4.5 Peptide mapping

S1.5 Set-up of enzymatic reactions
- S1.5.1 Substrate synthesis
- S1.5.2 Miniaturised enzymatic screening
- S1.5.3 Upscaling of reactions with substrate **1** and Cyc15 for product isolation and characterisation

S1.6 Crystallisation and structure elucidation of Cyc15
- S1.6.1 Protein crystallisation
- S1.6.2 Diffraction data collection
- S1.6.3 Docking studies

S2. Supplementary Figures

Figure S1. Structure of AbyU.
Figure S2. Three dimensional structures of selected cyclases described in the literature and putative spirotetronate cyclases.
Figure S3. Unrooted maximum likelihood phylogenetic analysis of protein sequences.
Figure S4. Docking results auf Cyc15 with product **2**.

S2.1 NMR spectral data
Table S1. Comparison summary of NMR data for AbyU product **2** (500 MHz) and isolated major Cyc15 product **3** (700 MHz).
Figure S5. Comparison of ^{1}H NMR data (500 MHz) of major Cyc15 product **3** and AbyU product **2** in $CDCl_3$.
Figure S6. Comparison of ^{13}C NMR data (125 MHz) of major Cyc15 product **3** and AbyU product **2** in $CDCl_3$.
Figure S7. ^{1}H NMR data (175 MHz) of major Cyc15 product **3** in $CDCl_3$.
Figure S8. ^{13}C NMR data (175 MHz) of major Cyc15 product **3** in $CDCl_3$.
Figure S9. 2D ^{1}H NMR ROESY data (600 MHz) of major Cyc15 product **3** in $CDCl_3$.

S2.2 UPLC-MS (QToF)
Figure S10. UPLC-MS (QToF) analyses of selected samples from the cyclase library screen with **1**.
Figure S11. UPLC-MS (QToF) data of the potential product peaks.

S3 Supplementary Schemes

Scheme S1. Displayed is a selection of natural polyketide natural products highlighting the cyclization steps in their biosynthetic pathways.

S4 Supplementary Tables

Table S2. Cyclases from the literature with proven or predicted spirotetronate-cyclase structural characteristics.
Table S3. Cyclases from the literature sharing between 24-97% sequence identity when aligned with Clustal Omega.
Table S4. Putative cyclases from the spirotetronate cyclase family selected from sequence similarity network analysis (2020-04).
Table S5. Non-spirotetronate cylclase-like cyclases selected from the literature.
Table S6. Summary of cyclase library information.
Table S7. Summary of X-ray data collection and refinement statistics.

S5 References

S1. Experimental Procedure

S1.1 Preparation of DNA constructs

The genes encoding for AbyU, AbmU, Sol5, and TedJ were obtained as previously described and cloned into the pOPINF vector using the In-Fusion™ system (Clontech).[1] The DNA fragments corresponding to AbmU, TedJ and Sol5 were PCR-amplified from commercially produced, codon optimised, synthetic DNA (Eurofins MWG) using the following primers:
AAGTTCTGTTTCAGGGCCCGATGAACGAACGGTTTACCTTACCGG (forward) and
TGGTCTAGAAAGCTTTA-TTACGCGGTACGTCCCGCA (reverse) for AbmU,
primers AAGTTCTGTTTCAGGGCCCGATGACTGATCCGGTGATCGTCGTTGG (forward) and
TGGTCTAGAAAGCTTTAGCGCGGGGTTCCGAACCAGTTC (reverse) for TedJ,
and primers AAGTTCTGTT-TCAGGGCCCGATGCGCTTCATCATTCTGAACTTACTGTC (forward) and
TGGTCTAGAAAGCTTTACACCAGTTTGAACCCACCTGGAACCAGT (reverse) for Sol5.
The PCR products were ligated into vector pOPINF[1b] using the In-FusionTM cloning system (Clontech). The resulting plasmids encoded N-terminally hexa-histidine-tagged proteins. The constructs were verified by DNA sequencing.
All other enzyme sequences (Table S2, S4-5) have been codon-optimised for *E. coli* expression and adjusted with SignalP 5.0[2] to the mature form of the protein when needed. Resulting sequences were synthesized and cloned by Twist Bioscience (South San Francisco, California, US) into the plasmid pET29b(+) utilising the restriction sites of NedI and XhoI.

S1.2 Sequence similarity network analysis

Based on the 14 cyclase sequences described in the literature which have proven or predicted spirotetronate-cyclase functional and/or structural characteristics (Table S2) BLAST based datamining was performed by which 286 non-redundant amino acid sequences were identify (2020-04). These sequences were evaluated by sequence similarity network (SSN) analysis. The SSN was built with the EFI-EST[3] platform with an alignment score threshold of 10 and visualized and analysed through Cytoscape 3.6.0.[4]
Amino acid sequences were aligned with MAFFT v7.490 and ran with the --auto flag.[5] A maximum-likelihood phylogenetic tree was produced with iqtree2 using ModelFinder Plus (MFP) and 1000 ultrafast bootstrap replicates.[6] The phylogenetic tree was visualized and annotated in FigTree v1.4.4 (https://github.com/rambaut/figtree).

S1.3 Homology modelling

Initial homology modelling was performed with Cyrus-CAD Bench application (CYRUS Biotechnology, Seattle, Washington State, United States) utilising the Rosetta protein design software (Table S4). Models have been evaluated if they contained as core structure the spirotetronate-cyclase typical eight-stranded β-barrel. Out of the selected 17 sequences nine did result in alternative structures. For these nine sequences homology models were built on the basis of available pdb entries of AbmU, AbyU, PyrI4, and Tsn15 with YASARA.[7] By this five of the initially 17 putative sequence have been identified to have very low probabilities to form the desired β-barrel core structure and were discarded for further investigations. With the release of AlphaFold 2.0[8] in 2021 new structural models have been predicted for the remaining 12 putative cyclases. Overall, the quality of the core structures has been improved yielding more defined substrate binding cavities. However prediction of the structure of the C- and N-terminal regions failed in 50% of the cases. On top of that three sequences have been predicted to form divergent structures compared to the eight-stranded β-barrel core. Of these three sequences two displayed similar structures compared to their initial homology modelling with Cyrus-CAD.

S1.4 Enzyme preparation

S1.4.1 Protein expression

All cyclases were recombinantly expressed in *E. coli* BL21 (DE3) using the same general procedure. Cell cultures harboring the transformed plasmids based on the pOPINF or pET29b(+) vector were grown in 500 mL ZYP-8012 auto induction media[9], supplemented with 50 µg/mL carbenicillin or 100 µg/mL kanamycin, respectively. Cells were grown at 37 °C with shaking at 200 rpm, until the optical density of the cultures at 600 nm had reached between 0.8-1.2. Cultures were grown for additional 16 h at 20 °C and subsequently harvested by centrifugation for 30 min at 4,500 x g, supernatant removed, and the remaining cell pellets stored at -80 °C.

S1.4.2 Protein purification with the streamlined protein supply process

12 cell pellets were thawed over night at 4 °C and 12 g (wet cell weight) were resuspended in 48 mL Buffer A (20 mM Tris-HCl, 150 mM NaCl, pH 7.5) supplemented with 0.6 g CellLytic™ Express (Sigma-Aldrich, Darmstadt, Germany) and incubated at room temperature for 30 min.

Furthermore for IMAC purification, 333 µL of Buffer B (20 mM Tris-HCl, 150 mM NaCl, 300 mM imidazole, pH 7.5) and approximately 1 mL of AmMag Ni magnetic beads (GenScript, New Jersey, US) were added to each sample. The mixture was incubated at 4 °C, under rotation for 90-120 min. A washing and one-step elution procedure was performed with the AmMag SA-1012 device (GenScript, New Jersey, US) using two washing steps each 10 mL with Buffer C (20 mM Tris-HCl, 150 mM NaCl, 2 mM imidazole, pH 7.5) and 4 mL Buffer B for the elution of each sample.

After a 20 min incubation time the supernatants were filtered through a 25 mm Acrodisc® Supor PF 0.8/0.2 µM membrane filter (Pall, California, US) and loaded onto two NGC chromatography systems (BioRad, Hercules, US) connected to a HiLoad™ 16/60 Superdex™ 200 prep grade (GE Healthcare, Chicago, US) column for size exclusion chromatography (SEC). Six samples per device were loaded automatically and eluted with Buffer A. Fractions containing the desired protein of interest, as determined by monitoring the absorbance of the column eluent at 280 nm followed by SDS-PAGE, were pooled and concentrated to a minimum of 1 mg/mL, and flash frozen in liquid nitrogen for storage at -80 °C.

When less than six samples were purified the method from Byrne et al[1a] was adopted using as IMAC wash buffer Buffer D (50 mM Tris-HCl, 150 mM NaCl, 20 mM imidazole, pH 7.5), as IMAC elution buffer Buffer E (50 mM Tris-HCl, 150 mM NaCl, 500 mM imidazole, pH 7.5) and as SEC buffer Buffer A.

S1.4.3 Protein melting temperature determination

The melting temperatures of the purified cyclases were determined by measuring their intrinsic fluorescence at 330 and 350 nm in presence of a heat gradient ranging from 35 to 95 °C (ΔT=3 °C/min) with a Tycho NT.6 (NanoTemper Inc., Munich, Germany). Approximately 10 µL of each sample containing 1 mg_{Prot}/mL was aspirated into standard-treated glass capillaries (Tycho NT. 6 TY-C001, NanoTemper Inc., München, Germany).

S1.4.4 Protein concentration determinations

Purified enzyme concentration was determined based on their predicted extinction coefficient and molecular weight (Table S6). Next, their absorbance at 280 nm was measured using a NanoDrop spectrophotometer (ND-1000, ThermoFisher Scientific, Waltham, US).

S1.4.5 Peptide mapping

First, 10 $µg_{Prot}$ of purified enzyme samples were worked up as previously described[10] to obtain reduced and alkylated peptide fragments through in-gel trypsin digestion. Resulting samples were analysed with the LC-MS system AdvanceBio 6546 XT LC/QToF, 1290 Infinity II (Agilent, Santa Clara, US) utilising the XBridge Peptide BEH C18 150 mm x 2.1 mm, 1.8 µm column (Waters Corp., Milford, US). The obtained MS/MS-data was analysed with the Agilent Mass Hunter BioConfirm 10.0 (Agilent, Santa Clara, US) software.

S1.5 Set-up of enzymatic reactions

S1.5.1 Substrate synthesis

The AbyU substrate analogue **1** was synthesised according to methods previously described.[1a]

S1.5.2 Miniaturised enzymatic screening

Reactions were performed in triplicates on 5 µL scale in a 384-well high recovery well microplate (384 Well Microplate, PP, V-Bottom, #781280, Greiner Bio-One, Kremsmünster, Austria) covered with a silicon seal. Incubations were performed at 25 °C and 600 rpm in a ThermoMixer with ThermoTop (Eppendorf, Hamburg, Germany). 4.5 µL reaction mixture were dispensed into the plate containing 50 mM Tris-HCl, 150 mM NaCl, pH 7.5, and 1 mg_{Prot}/mL cyclase. For control reactions no enzyme was added to the buffer solution. Reactions were started by addition of 0.5 µL 10 mM substrate solution in acetonitrile (final concentration 1 mM substrate, 10% (v/v) acetonitrile) which was dispensed with a Mosquito® HV (SPT Labtech, Melbourn, UK). Reactions were quenched with 20 µL acetonitrile, sealing the plate using a thermal heat sealer (Velocity11´s PlateLoc, Agilent Technologies, Santa Clara, US), mixing the samples for 1 min at 1200 rpm, and centrifuging for 15 min at 3000 x g (Eppendorf 5810 R, Eppendorf, Hamburg, Germany). 10 µL of sample were injected in a Waters Acquity Ultra-Performance Liquid Chromatography (UPLC) system paired with a SYNAPT G2 high definition (HDMS) quadrupole time-of-flight (QToF) mass spectrometer (Waters Corp., Milford, US). Electrospray ionization data were obtained in positive ion mode (ES+). All data were acquired in MS^E mode, which allows the exact mass determination for both the precursor and fragment ions in a single analysis by performing the acquisition at low- and high-energy, respectively. Mobile phases were 0.1% formic acid in water (A) and acetonitrile (B). Samples were injected into an Acquity UPLC BEH-C18 column (130 Å, 1.7 µm, 2.1 mm x 100 mm, Waters Corp., Milford, US). The gradient was: 10-99% B,

8 min with an isocractic step at 52% B for 5 min. Flow rate was 0.5 mL/min. Leucine enkephalin was used for lock mass at a concentration of 2 ng/µL in 0.1% (v/v) formic acid: acetonitrile (1:1). Data acquisition and processing were performed using MassLynx v4.2 (Waters Corp., Milford, US).

S1.5.3 Upscaling of reactions with substrate 1 and Cyc15 for product isolation and characterisation

To investigate the major product formed in the reaction of Cyc15 with **1**, first the reaction was performed with 13.4 mg substrate (38.9 µmol). 39 mL reaction mixture (containing 1 mM **1**, 10% (v/v) acetonitrile, 1 mg_{Prot}/mL Cyc15, 20 mM Tris-HCl, 150 mM NaCl, pH 7.5) were incubated in a 250 mL Erlenmeyer flask at 25 °C and 200 rpm for 3 h. The reaction was extracted three times with 65 mL ethyl acetate and the collected organic phases were dried over magnesium sulfate, the desiccant filtered off and the solvent evaporated under reduced pressure. The residue was dissolved in acetonitrile (1 ml) prior to semi-preparative HPLC purification using a Waters 2445SFO HPLC system (Waters Corp., Milford, US) with a Waters 2298 diode array detector for UV between 200 and 400 nm. The system was equipped with a Phenomenex LUNA column (5 µm, C18, 100 Å, 4.6 × 250 mm; Phenomenex, Torrance, US) and was eluted with a linear gradient of 50-90% acetonitrile in water with 0.05% formic acid; flow rate: 1 mL/min; 20 min, detection: 254 nm. The product was collected at t_R = 9.4 min allowing an analytical sample of the proposed Diels-Alder product **3** to be isolated. The sample was dried in vacuo and subsequently under a stream of nitrogen. The dry samples was dissolved in chloroform-d and subjected to ^{1}H NMR analysis on a Bruker Avance III HD 700 spectrometer (Bruker, Billerica, US).

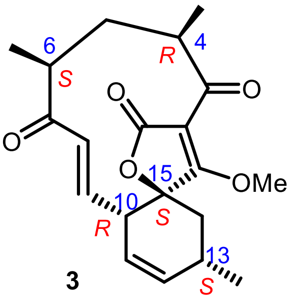

3

H NMR (700 MHz, $CDCl_3$) 6.57 (1H, d, *J* 16.0, 8-H), 6.37 (1H, dd, *J* 16.0, 9.5, 9-H), 5.92 (1H, dt, *J* 10.0, 3.0, 11-H), 5.66 (1H, dt, *J* 10.0, 2.5, 12-H), 3.82 (3H, s, OCH_3), 3.34 (1H, *app.* dq, *J* 9.0, 3.0, 10-H), 2.63 (1H, m, 6-H), 2.45 (1H, m, 13-H), 2.27 – 2.20 (2H, m, 5-*H*H, 4-H), 2.14 (1H, dd, *J* 14.0, 5.5, 14-*H*H), 1.67 (1H, dd, *J* 14.0, 8.0, 14-H*H*), 1.30 (1H, m, 5-H*H)*, 1.20 (3H, d, *J* 7.0, 6-CH_3), 1.18 (3H, d, *J* 6.0, 4-CH_3), 1.14 (3H, d, *J* 7.0, 13-CH_3). C NMR (175 MHz, $CDCl_3$) 204.7 (C-7), 199.9 (C-4), 177.8 (C-16), 169.4 (C-3), 142.0 (C-9), 137.6 (C-11), 130.8 (C-8), 124.9 (C-12), 106.9 (C-2), 86.6 (C-15), 61.0 (OCH_3), 50.4 (C-4), 47.4 (C-6), 45.4 (C-10), 38.3 (C-14), 35.7 (C-5), 28.3 (C-13), 20.4 (13-CH_3), 16.7 (6-CH_3), 15.0 (4-CH_3).

S1.6 Crystallisation and structure elucidation of Cyc15

S1.6.1 Protein crystallisation

Cyc15 for crystallisation experiments was produced and purified as previously described (Section S1.4.1 and S1.4.2). The protein expression procedure was altered by utilising LB media and inducing expression with 1 mM IPTG when cells have grown to OD_{600nm} of 0.5. The purification method according to the one described for less than six samples was applied. Conditions to support the crystal formation of Cyc15 were identified by employing commercially available crystallisation screens from Molecular Dimensions (Sheffield, UK) utilising the sitting drop vapor diffusion method at 20 °C. The screens were set up in 96-well-plates (MRC 2 LENS plate from Swiss Ci, High Wycombe, UK), suspending 50 µL of the screen conditions into the reservoirs, and dispensing with a Nanodispenser Mosquito® Xtal3 (SPT Labtech, Melbourn, UK) 150 nL or 175 nL reservoir solution and 150 nL or 125 nL protein solution (10 mg/mL), respectively. Diffraction quality crystals were grown in a condition containing 100 mM glutamate monohydrate, 100 mM DL-alanine; 100 mM glycine; 100 mM DL-lysine monohydrochloride; 100 mM DL-serine; 100 mM imidazole, 100 mM MES monohydrate (acid), pH 6.5; 20% (v/v) ethylene glycol, 10% (w/v) PEG 8000. Crystals took up to six weeks to grow.

S1.6.2 Diffraction data collection

Crystals selected for diffraction data collection were soaked in 30% glycerol as a cryoprotectant, then mounted in appropriately sized litholoops (Molecular Dimensions Ltd) and flash-cooled in liquid nitrogen prior to analysis. Diffraction data were collected at Diamond Light Source, UK, on beamline I04 using a Dectris Eiger2 XE 16M pixel array detector. Diffraction data was auto-processed by the Diamond Light Source Automatic Software Pipeline using xia2 3dii.[11] 5% of the data were set aside for the calculation of R_{free}. The resulting data was used to solve the structure of Cyc15 using the CCP4i2 suite (version 7.1) of programs.[12] The structure of Cyc15 was determined by molecular replacement using MOLREP[13], using as a search model a Cyc15 homology model obtained from AlphaFold 2.0.[8] Iterative rounds of manual model building and refinement using COOT[14] and Refmac5[15] were used to refine the structure (pdb code: 8OF7). Data collection, phasing and refinement statistics for Cyc15 are provided in Table S7. Protein structure graphics were prepared using PyMOL Molecular Graphics System, Version 2.0 Schrödinger, LLC.

S1.6.3 Docking studies

Molecular docking of the bicyclic products **2** and **3** of Cyc15 was performed using the crystal structure complexed with PEG. Chain A was used and all molecules except the glycine (Gly3401) molecule bound in the active site were removed. The Maestro (Schrödinger, LLC, New York, NY, 2021) suite was utilised to (i) first prepare the protein structure with the "Protein Preparation Wizard", (ii) subsequently the refined structure was used to prepare the grid for docking with the "Receptor Grid Generation" tool of the Glide toolbox,[16] (iii) next the ligands were prepared with the "LigPrep" tool to determine their chiralities from 3D structure and (iv) last the "Glide Ligand docking" was performed, in which the glycine molecule was removed and a maximum output of the 20 best structures was set. All preparative calculations were run for conditions at pH 7.5.

S2. Supplementary Figures

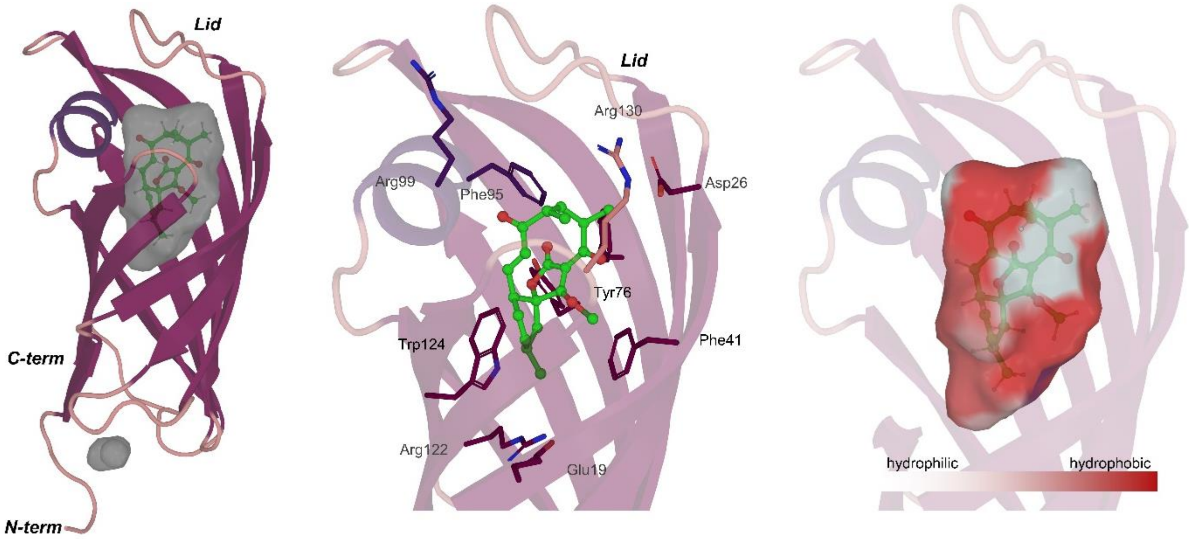

Figure S1: Structure of AbyU in the closed lid-conformation (magenta) with the modelled *O*-methylated AbyU product (**2**, green sticks).[1a, 17] Key residues in the active site have been displayed as sticks and the surface of the cavity has been coloured in grey (left) and according to its hydrophobicity (right) from hydrophilic (white) to hydrophobic (red).

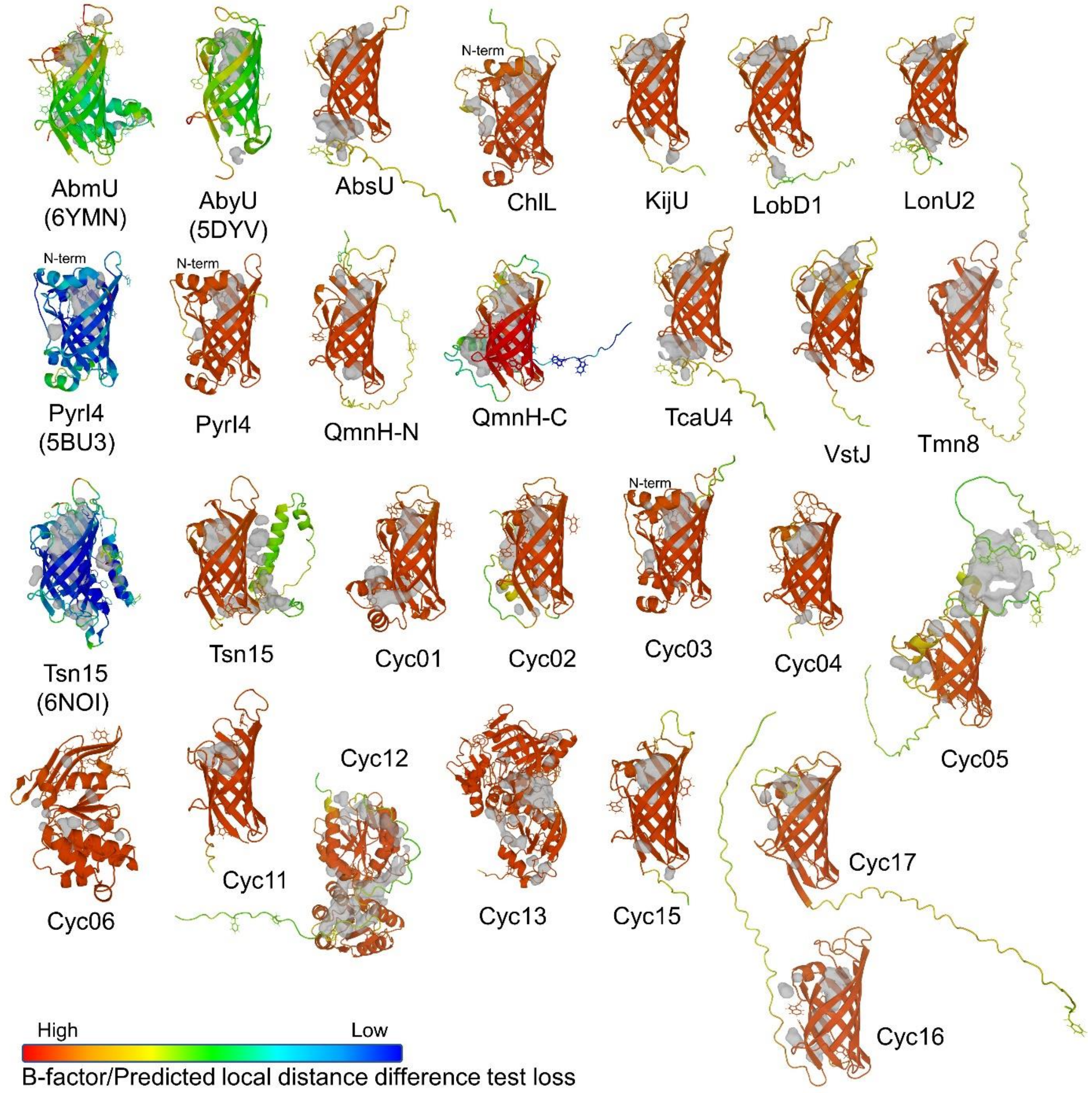

Figure S2. Three dimensional structures of selected cyclases described in the literature and putative spirotetronate cyclases. Displayed are the AlphaFold 2.0 models which are coloured according to the confidence of the model (Predicted local distance difference test) from high (red) to low (blue). Available crystal structures of AbmU (6YMN), AbyU (5DYU), PyrI4 (5BU3), and Tsn15 (6NOI) have been coloured corresponding to their flexibility (B-factor) from high (red) to low (blue).

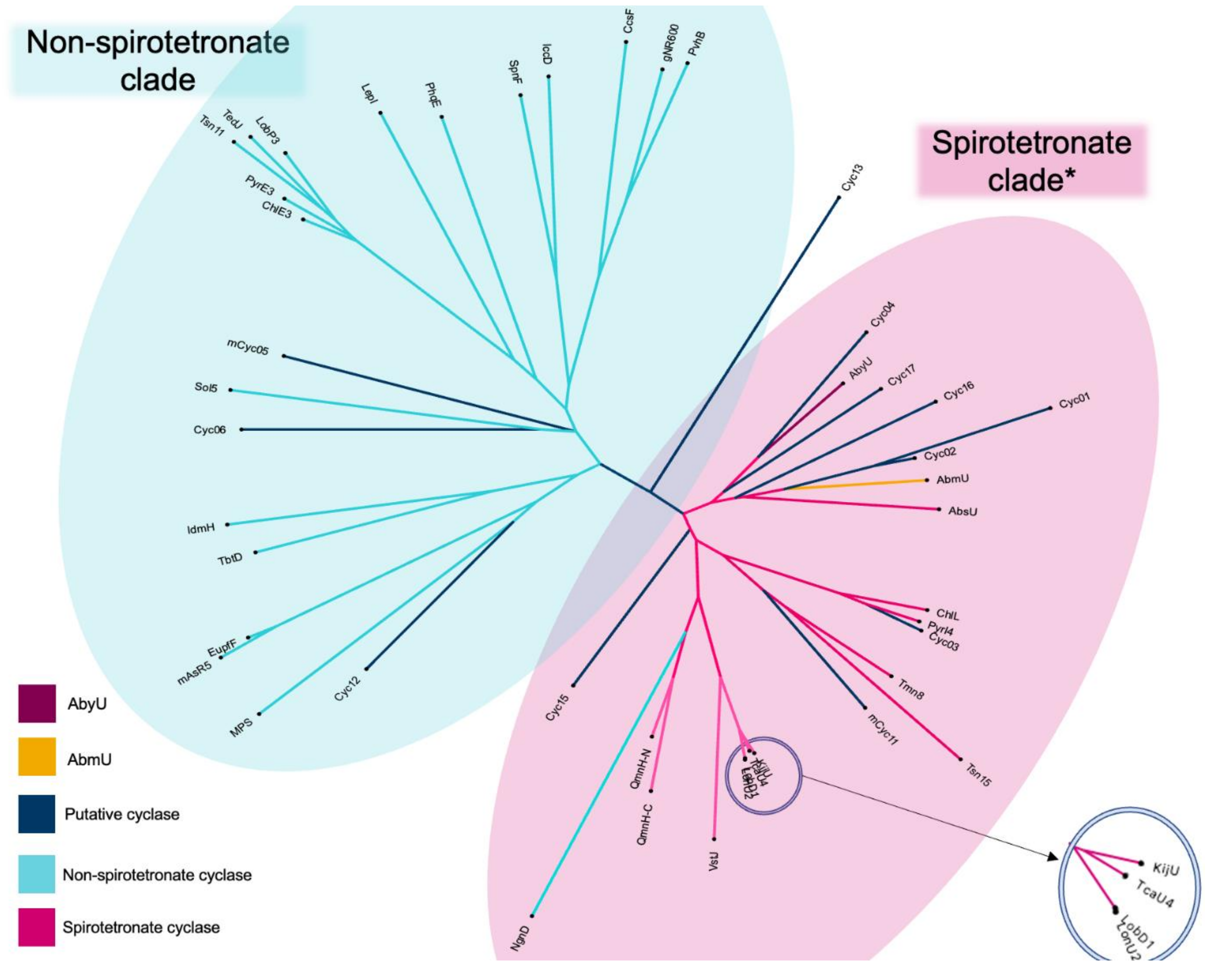

Figure S3. Unrooted maximum likelihood phylogenetic analysis of protein sequences. IQ-tree2 MFP selected WAG+F+R4 as the best model and identified 152 constant sites (15.46%) across the 45 sequences, Branches coloured according to proven or predicted spirotetronate activity (dark-blue), non-spirotetronate cyclases (light-blue) and putative cyclases from this study (pink). *****We recognize that NgnD, a non-spirotetronate enzyme, is included in the 'spirotetronate' clade. NgnD may have reverted to a non-spirotetronate cyclase or been incorrectly assigned in the tree. The clade structure has been maintained in the figure for clarity.

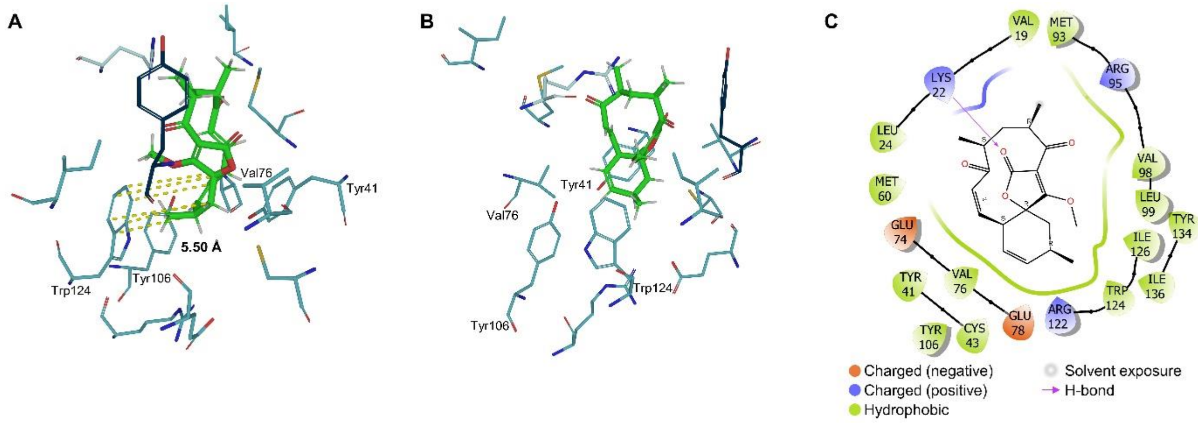

Figure S4. (**A**&**B**) Docked product **2** (green) from two perspectives is displayed in concert with the active site residues, highlighting the distance (yellow) of the cyclohexene ring towards Trp124. Crystal structure of Cyc15 Chain A (8OF7) displayed are residues of the active site highlighted and labelled as blue sticks and (**C**) assigned from the docking studies to interact with product **2** binding.

S2.1 NMR spectral data

Table S1. Comparison summary of NMR data for AbyU product **2** (500 MHz) and isolated major Cyc15 product **3** (700 MHz) in $CDCl_3$.

Position	Type	AbyU product analogue 2[1a]		Major Cyc15 product 3	
		δ_H (ppm)	δ_C (ppm)	δ_H (ppm)	δ_C (ppm)
**1**	quat.	–	169.9	–	169.4
**2**	quat.	–	106.9	–	106.9
**3**	quat.	–	200.6	–	199.9
**4**	-CH	3.11, m	46.63	2.23, overlapping m	50.4
**5**	$-CH_2$	1.86, ddd, *J* 15.0, 6.0, 4.0	38.9	2.27 – 2.20, m	35.7
		1.18, m		1.30, m	
**6**	-CH	2.94, sextet, *J* 6.5	46.57	2.63, m	47.4
**7**	quat.	–	204.3	–	204.7
**8**	-CH	6.24, d, *J* 16.5	131.6	6.57, d, *J* 16.0	130.8
**9**	-CH	6.46, dd, *J* 16.5, 7.0	141.5	6.37, dd, *J* 16.0, 9.5	142.0
**10**	-CH	3.44, m	44.6	3.34, *app.* dq, *J* 9.0, 3.0	45.4
**11**	-CH	5.67, *app*. dt, *J* 10.0, 3.0	121.8	5.92, dt, *J* 10.0, 3.0	137.6
**12**	-CH	5.85, *app*. dt, *J* 10.0, 3.0	136.7	5.66, dt, *J* 10.0, 2.5	124.9
**13**	-CH	2.63, m	29.3	2.45, m	28.3
**14**	$-CH_2$	2.39, dd, *J* 14.5, 8.0	36.6	2.14, dd, *J* 14.0, 5.5	38.3
		1.81, dd, *J* 14.5, 4.5		1.67, dd, *J* 14.0, 8.0	
**15**	quat.	–	86.0	–	86.6
**16**	quat.	–	178.2	–	177.8
**17**	$-CH_3$	1.14, d, *J* 7.5	21.1	1.14, d, *J* 7.0	20.4
**18**	$-CH_3$	1.18, d, *J* 6.5	16.6	1.18, d, *J* 6.0,	15.0
**19**	$-CH_3$	1.20, d, *J* 7.0	17.0	1.20, d, *J* 7.0	16.7
**20**	$-CH_3$	3.90, s	61.7	3.82, s	61.0

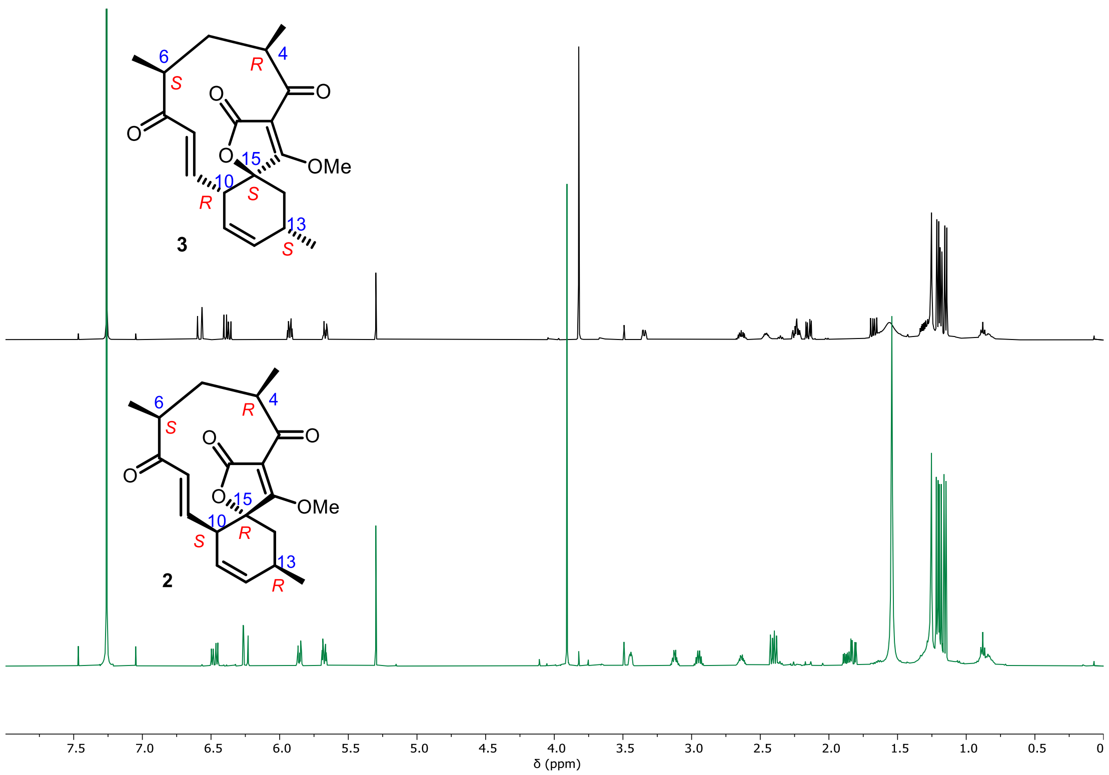

Figure S5. Comparison of ^{1}H NMR data (500 MHz) of major Cyc15 product **3** and AbyU product **2** in $CDCl_3$.

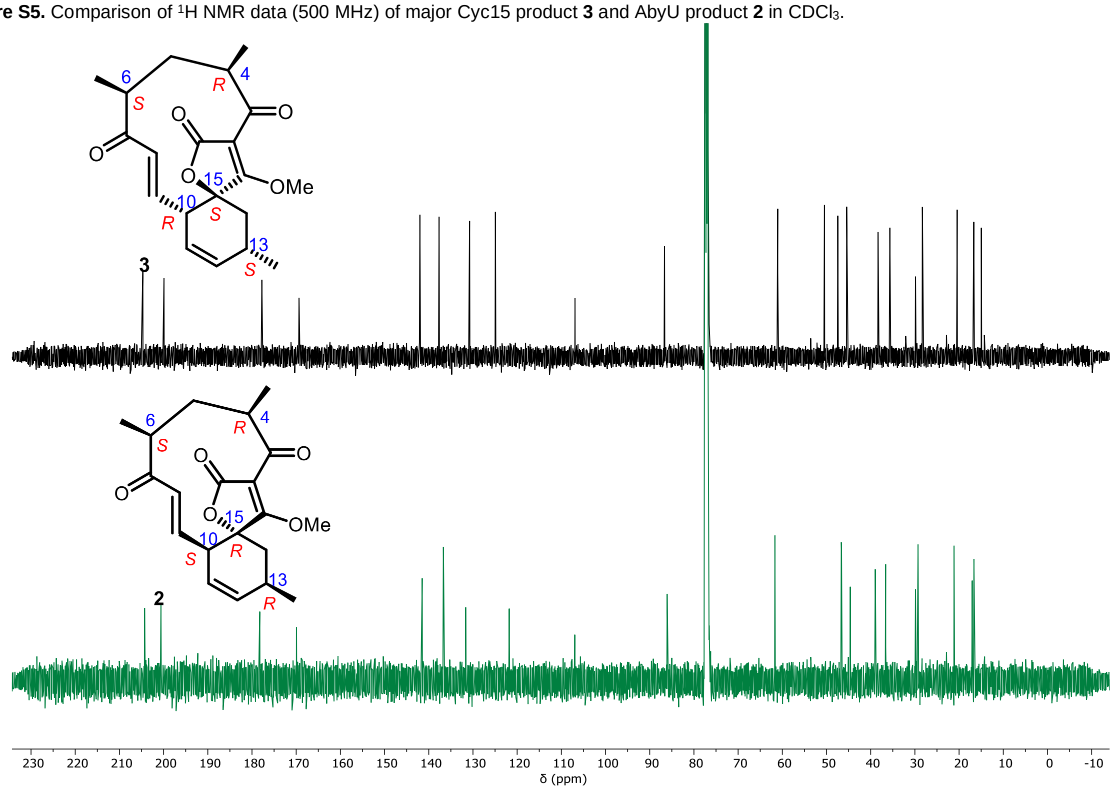

Figure S6. Comparison of ^{13}C NMR data (125 MHz) of major Cyc15 product **3** and AbyU product **2** in $CDCl_3$.

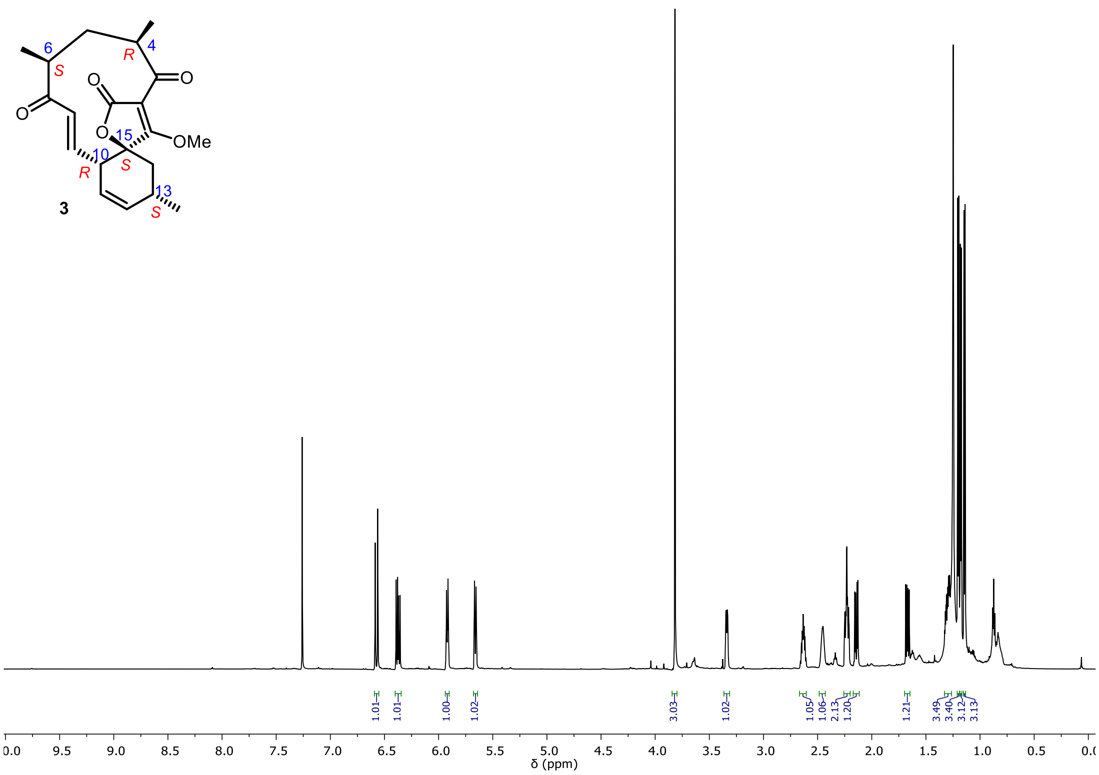

Figure S7. ^{1}H NMR data (700 MHz) of major Cyc15 product **3** in $CDCl_3$.

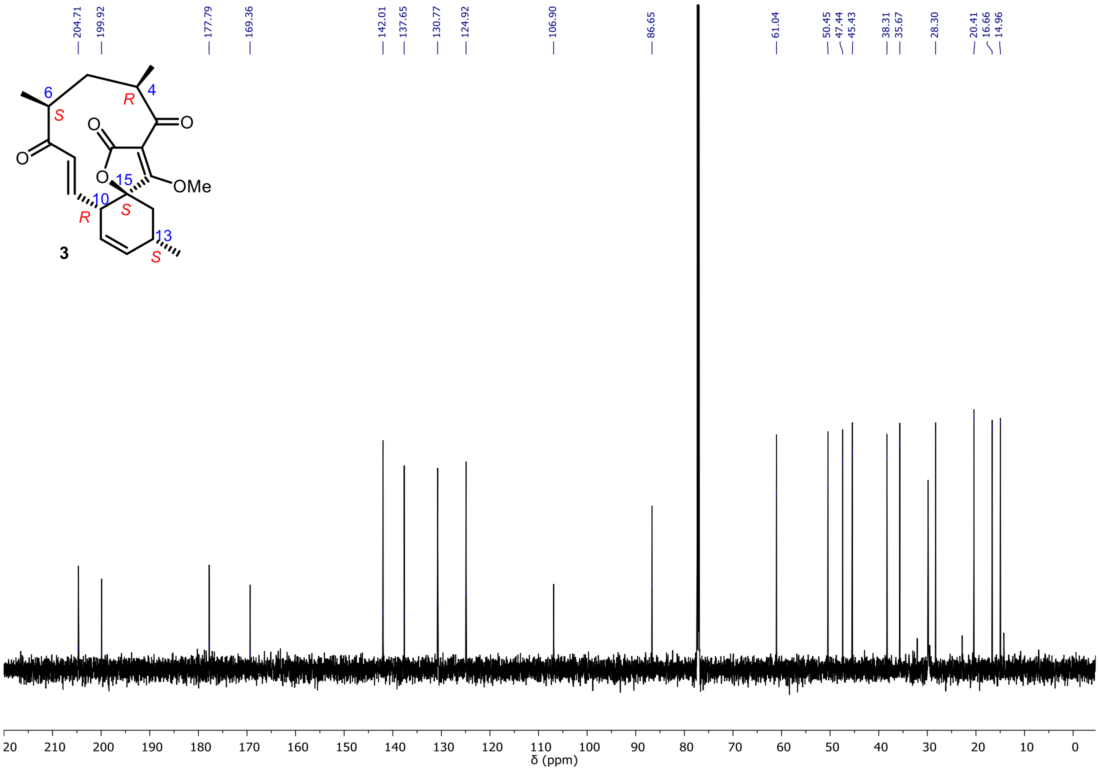

Figure S8. ^{13}C NMR data (175 MHz) of major Cyc15 product **3** in $CDCl_3$.

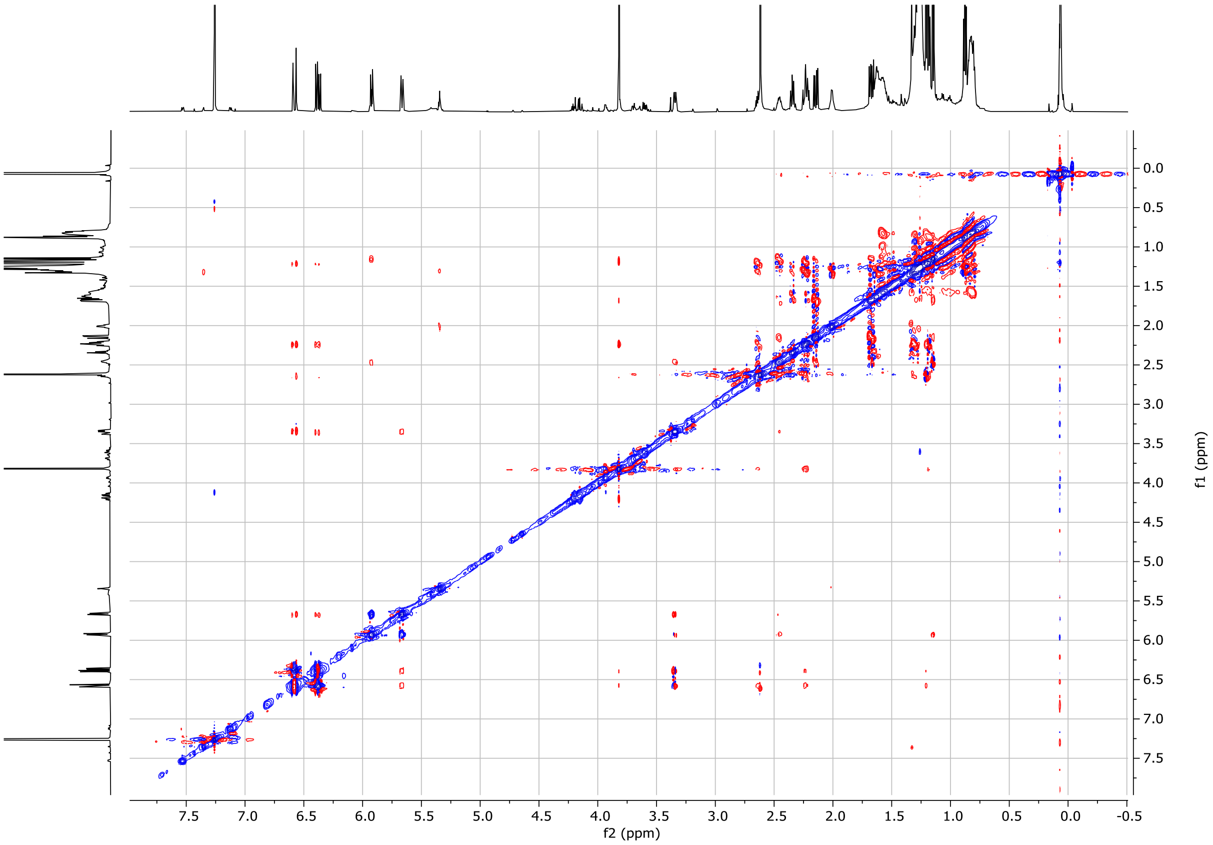

Figure S9. 2D ^{1}H NMR ROESY data (600 MHz) of major Cyc15 product **3** in $CDCl_3$.

S2.2 UPLC-MS (QToF)

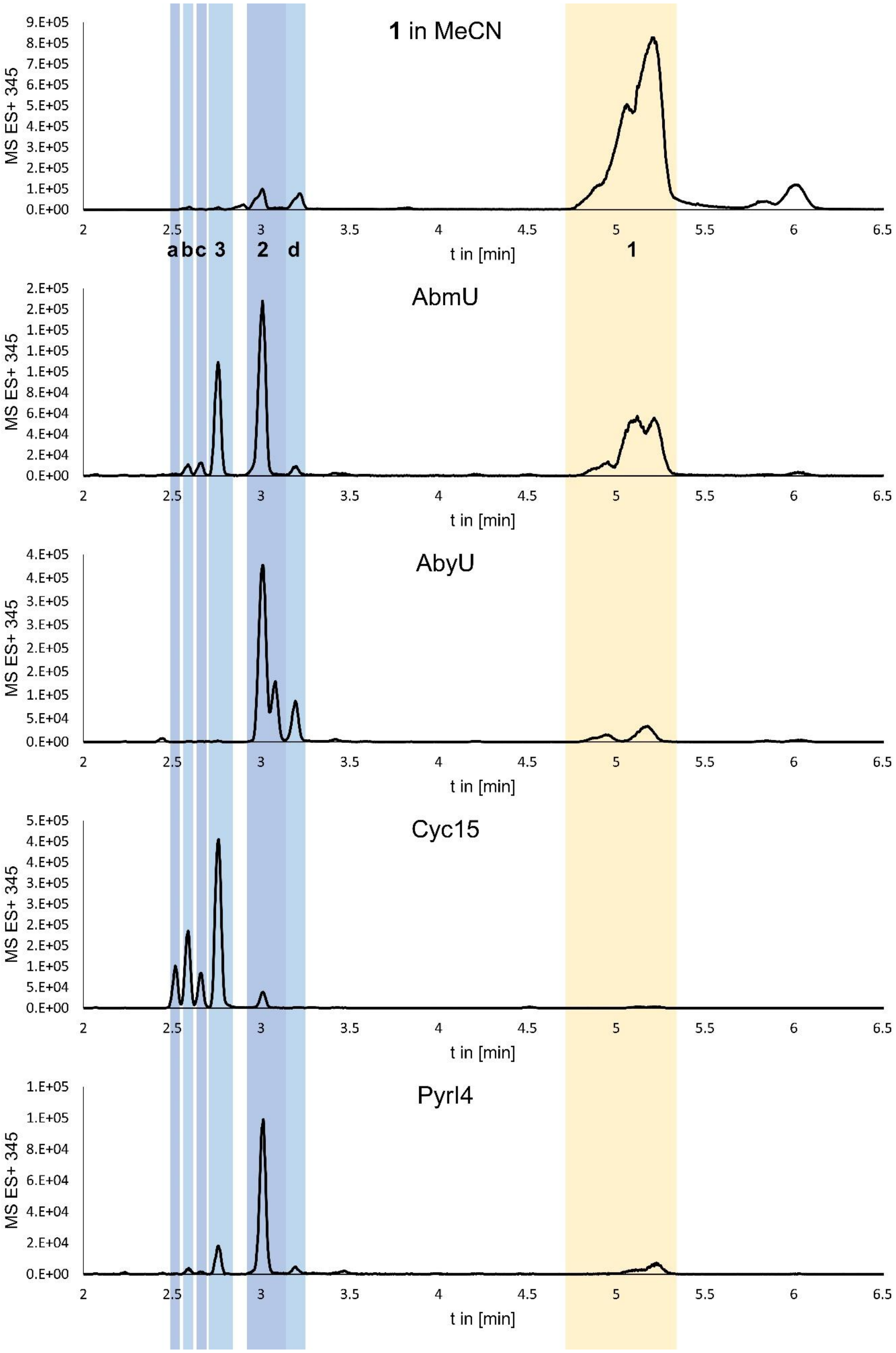

Figure S10. UPLC-MS (QToF) analyses of selected samples from the cyclase library screen with **1** after 20 min incubations. Displayed is the ion current resulting from the extracted mass of the total ion current of the substrate (**1**; yellow; t_R = 5.24 min) and products (blue: **3**, t_R = 2.76 min; **2**, t_R = 3.02 min; products **a-c**, t_R = 2.48-2.70 min, product **d**, t_R = 3.16) masses which are equal to $[M+H]^+$ 345.170 *m/z*.

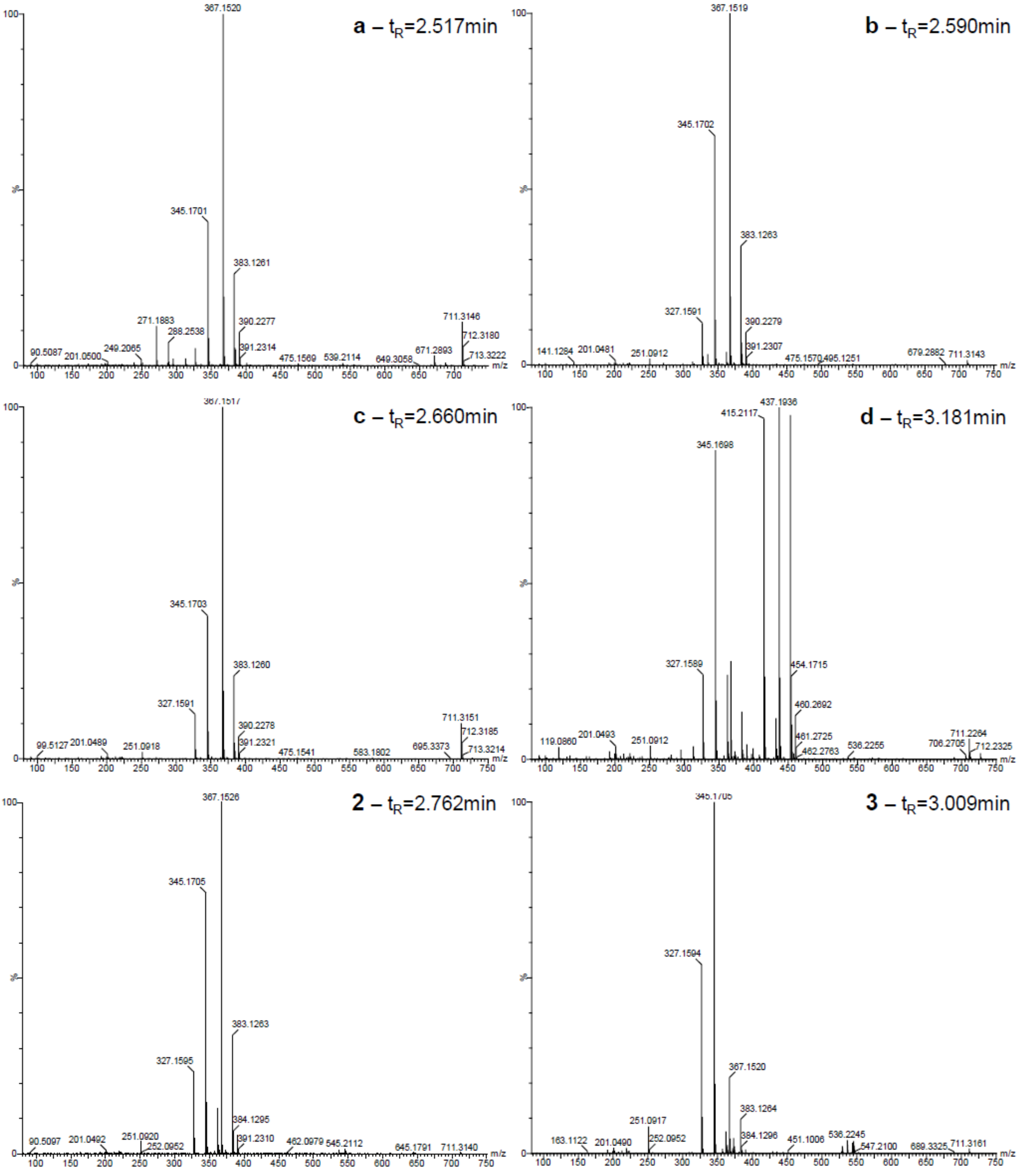

Figure S11. UPLC-MS (QToF) data of the potential product peaks (**a-c**, t_R = 2.48-2.70 min; **d**, t_R = 3.16; **3**, t_R = 2.76 min; **2**, t_R = 3.02 min) confirming the expected *m/z* of $[M+H]^+$ 345.170 *m/z* and $[M+Na+H]^+$ 367.152 *m/z*.

S3 Supplementary Schemes

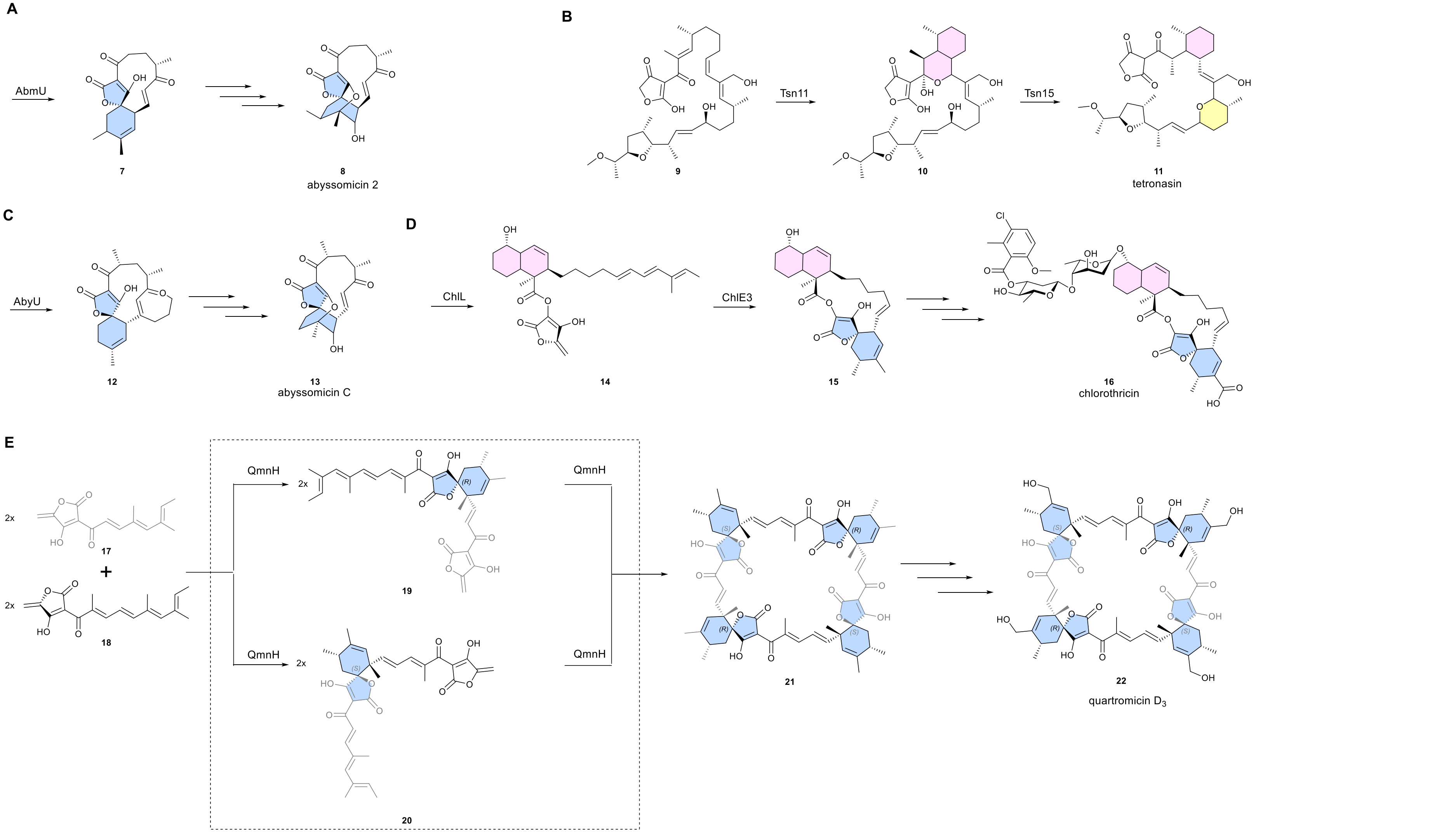

Scheme S1. Displayed is a selection of natural polyketide natural products highlighting the cyclization steps in their biosynthetic pathways. This includes [4+2]-spirocyclisations (blue), Diels-Alder type decalin ring formation (pink) and a polycyclic rearrangement to tetronasin (yellow). Spirotetronate cyclases AbmU, AbyU, ChlL, and QmnH are involved in the biosynthesis of abyssomicin 2 (**A**), abyssomicin C (**C**), chlorothricin (**D**), and quatromicin D_3 (**E**), respectively and they are followed by further tailoring enzymes to form the biosynthetic products. Decalin-ring forming enzymes are involved in the biosynthesis of chlorothricin (ChlE3, **D**) and tetronasin (Tsn11, **B**). In the latter biosynthesis, the final product is obtained by a subsequent polycyclic rearrangement which is catalysed by Tsn15.

S4 Supplementary Tables

Table S2. Cyclases from the literature sharing sequence homology between 24-97% sequence identity with proven or predicted spirotetronate-cyclase structural characteristics.

Cyclase Name	***Origin/ Organism***	Genbank #	Natural product	Reference
AbmU	*Streptomyces koyangensis*	MG243704.1<br>(6LE0, 6YMN)	(Neo-)Abyssomicins	[18]
AbyU	*Micromonospora maris (strain AB-18-032)*	CP002638.1<br>(pdb: 5DYQ, 5DYV)	Abyssomicin C	[1a]
AbsU	*Streptomyces* sp. LC-6-2	ARE67838	Abyssomicins	[19]
ChlL	*Streptomyces antibioticus*	AAZ77701	Chlorothricins	[20]
KijU	*Actinomadura kijaniata* SCC1256 (ATCC 31588)	WP_157420330.1	Kijanimicin	[21]
LobD1	*Streptomyces* sp. FXJ7.023	L7RSB5	Lobophorins	[22]
LonU2 (LobU2)	*Streptomyces* sp. SCSIO 01127	AGI99498.1	Lobophorins	[23]
PyrI4	*Streptomyces rugosporus*	K7QVW7<br>(pdb: 5BTU)	Pyrroindomycins	[24]
QmnH-C	*Amycolatopsis orientalis*	K4FDH3 res1-178	Quartromicin	[25]
QmnH-N	*Amycolatopsis orientalis*	K4FDH3 res179-376	Quartromicin	[25]
TcaU4	*Micromonospora chalcea*	ACB37739	Tetrocarcin A	[26]
Tmn8	*Streptomyces* sp. NRRL 11266	BAF73716	Tetronomycin	[27]
VstJ	*Streptomyces versipellis*	A0A0B6VRF8	Versipelostatin	[28]
Tsn15	*Streptomyces longisporoflavus*	WP_147879045.1<br>(pdb: 6NOI, 6NNW)	Tetronasin	[29]

Table S3. Cyclases from the literature sharing between 24-97% sequence identity when aligned with Clustal Omega[30] and have proven or predicted spirotetronate-cyclase structural characteristics.

	AbmU	AbsU	AbyU	ChlL	KijU	LobD1	LonU2	PyrI4	QmnH-C	QmnH-N	TcaU4	Tmn8	Tsn15	VstJ
AbmU		24.4%	24.7%	22.0%	19.3%	18.5%	18.0%	21.6%	22.2%	23.1%	18.0%	21.8%	16.2%	20.8%
AbsU	24.4%		21.4%	17.3%	21.9%	18.4%	19.0%	14.0%	18.1%	22.2%	21.1%	16.6%	17.6%	18.2%
AbyU	24.7%	21.4%		12.9%	22.9%	21.4%	21.4%	15.1%	19.9%	25.9%	22.2%	19.2%	11.1%	21.1%
ChlL	22.0%	17.3%	12.9%		20.1%	18.2%	18.2%	44.1%	18.5%	16.2%	19.5%	18.0%	14.2%	18.3%
KijU	19.3%	21.9%	22.9%	20.1%		70.2%	71.5%	20.8%	23.0%	27.6%	75.0%	20.9%	15.2%	31.9%
LobD1	18.5%	18.4%	21.4%	18.2%	70.2%		97.4%	20.1%	19.3%	24.6%	69.5%	15.5%	15.9%	34.7%
LonU2	18.0%	19.0%	21.4%	18.2%	71.5%	97.4%		19.5%	19.3%	24.0%	69.5%	15.5%	15.9%	34.0%
PyrI4	21.6%	14.0%	15.1%	44.1%	20.8%	20.1%	19.5%		16.1%	17.7%	19.0%	17.6%	16.1%	21.8%
QmnH-C	22.2%	18.1%	19.9%	18.5%	23.0%	19.3%	19.3%	16.1%		36.1%	22.7%	19.5%	14.0%	17.1%
QmnH-N	23.1%	22.2%	25.9%	16.2%	27.6%	24.6%	24.0%	17.7%	36.1%		25.1%	16.9%	11.6%	21.2%
TcaU4	18.0%	21.1%	22.2%	19.5%	75.0%	69.5%	69.5%	19.0%	22.7%	25.1%		18.8%	14.2%	32.6%
Tmn8	21.8%	16.6%	19.2%	18.0%	20.9%	15.5%	15.5%	17.6%	19.5%	16.9%	18.8%		25.6%	24.2%
Tsn15	16.2%	17.6%	11.1%	14.2%	15.2%	15.9%	15.9%	16.1%	14.0%	11.6%	14.2%	25.6%		14.8%
VstJ	20.8%	18.2%	21.1%	18.3%	31.9%	34.7%	34.0%	21.8%	17.1%	21.2%	32.6%	24.2%	14.8%	

Table S4. Putative cyclases from the spirotetronate cyclase family selected from sequence similarity network analysis (2020-04). Genomic data was analysed with antiSMASH 6.1.1[31] (2022-10) identifying related to the location of the putative cyclase's coding regions (CDS) the similarity to other biosynthetic gene clusters (BGC). Using different homology modelling (HM) tools the likelihood of the putative cyclases exhibiting the typical eight-stranded β-barrel core structure was evaluated alongside their ability to be recombinantly expressed in *E. coli* under non optimised conditions. Expressed and purified enzymes were tested for their cyclase activity using substrates **1**. (Yes – cyclase exhibited desired feature, X – cyclase didn't exhibit desired feature, - - was not analysed due to previous results obtained)

Cyclase Name	*Origin/ Organism*	Genbank #	Genome	Genome region (CDS)	Similar BGC	HM Cyrus CAD	HM YASARA	HM AF2	Expres-sion	Activity
Cyc01	*Erwinia persicina*	TKJ90061.1	QGAC01000010.1	164864-5373	Ustilagic acid, *ustilago maydis* 251	Yes	-	Yes	X	-
Cyc02	*Streptomyces albofaciens*	WP_150242566.1, KAA6223876.1	NZ_PDCM01000001.1	4481168-692	Kistamicin A (28%)	Yes	-	Yes	X	-
Cyc03	*Nonomuraea polychroma*	WP_127931359.1, RVX38772.1	NZ_SAUN01000001.1	1239590-40162	Chlorothricin (41%)	Yes	-	Yes	Yes	X
Cyc04	*Streptomyces varsoviensis*	WP_030882074.1	LGUT01000218.1	1552-974	No results	Yes	-	Yes	Yes	Yes
mCyc05	*Gossypium raimondii*	XP_012455938.1	CM001750.1	57197326-8045	No results	X	Yes	Yes	Yes	Yes
Cyc06	*Chitinophagaceae bacterium*	RYY57898.1	SDZZ01000423.1	350-970	No results	X	Yes	X	X	Yes
Cyc07	*Gemmataceae bacterium*	HGX06851.1	DSRJ01000398.1	1169-2569	No results	X	X	-	-	-
Cyc08	*Gemmatimonadetes bacterium*	PYP72993.1	QHVD01000011.1	8757-9146	No results	X	X	-	-	-
Cyc09	*Succiniclasticum ruminis*	SDC07064.1	FMYW01000002.1	123167-5137	No results	X	X	-	-	-
Cyc10	*Salpingoeca rosetta*	XP_004991816	GL832972.1	84355-122474	No results	X	X	-	-	-
mCyc11	*Gammaproteobacteria bacterium*	HED19221.1	DRJT01000345.1	4116-4568	No results	Yes	-	Yes	Yes	Yes
Cyc12	*Porphyrobacter* sp. LM 6	AOL95741.1	CP017113.1	2906747-7880	No cluster	X	Yes	X	Yes	Yes
Cyc13	*Deltaproteobacteria bacterium* GW2_57_13	OGP21310.1	MGPN01000202.1	6056-7379	No results	X	Yes	X	Yes	Yes
Cyc14	*Mesorhisobium* sp.	RWJ97006.1	SAOY01000061.1	19816-20364	No results	X	X	-	-	-
Cyc15	*Streptomyces* sp. NL15-2K	WP_124445685.1	NZ_BHXA01000189.1	16573-7010	Versipelostatin (22%)	Yes	-	Yes	Yes	Yes
Cyc16	*Amycolatopsis pithecelloba*	WP_154760925.1	NZ_WMBA01000079.1	18474-9013	Chlorothricin (16%)	Yes	-	Yes	X	-
Cyc17	*Actinocrispum wychmicini*	WP_132116074.1	NZ_SLWS01000003.1	508997-509545	Chlorothricin (48%)	Yes	-	Yes	X	-

Table S5. Non-spirotetronate cylclase-like cyclases selected from the literature.

Cyclase Name	*Origin/ Organism*	Genbank #	Natural product	Reference
CcsF	*Aspergillus clavatus*	ACLA_78690	Cytochalasin E	[32]
ChlE3	*Streptomyces antibioticus* DSM 40725	AAZ77700.1	Chlorothricins	[20]
EupfF	*Penicillium janthinellum*	USQ93120.1	Neosetopophomone B & Eupenifeldin	[33]
gNR600	Fungal sp. (strain NRRL 50135)	AIP87501	Equisetin	[34]
IccD	*Cylindrocladium ilicicola* MFC-870, *Talaromyces variabilis (Penicillium variabile)*	QBQ83708.1	Ilicicolin H	[35]
ImdH	*Streptomyces antibioticus* NRRL 8167	ACN69984.1 (pdb: 6HNN, 6HNL, 6HNM)	Indamycin	[36]
LepI	*Apergillus nidulans & flavus*	EED49872.1 (pdb: 6IX3, 6IX5, 6IX7-9)	Leporin C	[37]
LobP3	*Streptomyces* sp. SCSIO 01127	AGI99493.1	Lobophorins	[22]
NgnD	*Nocardia argentinensis* ATCC 31306	AXG22404.1 (pdb: 6A5F)	Nargenicin	[38]
mAsR5	*Sarocladium schorii* (formerly *Acremonium strictum* IMI 501407))	AWM95794	Xenovulene A	[39]
MPS	*Macrophoma commelinae*	BAA89352.1 (pdb: 1IZC)	Macrophomate	[40]
PhqE	*Penicillium fellutanum*	AGA37272.1 (pdb: 6NKI, 6NKK, 6NKM)	Paraherquamides	[41]
PvhB	*Penicillium variabile (Talaromyces variabilis)*	AZZ09608.1, AZZ09615.1	Varicidin A	[42]
PyrE3	*Streptomyces rugosporus*	AFV71312.1 (pdb: 5XGV)	Pyrroindomycins	[24]
Sol5	*Alternaria solani, Ascochyta rabiei*	AB514562 AIU56804.1	Solanapyrones	[43]
SpnF	*Saccharopolyspora spinosa*	AAG23267.1 (pdb: 4PNE)	Spinosyn A	[44]
TbtD	*Thermobispora bispora*	ADG87279.1 (pdb: 5WA3)	Thiomuracin	[45]
TedJ	*Streptomyces* sp. LC-6-2	WP_062758178.1	Tetrodecamycins	[46]
Tsn11	*Streptomyces longisporoflavus*	ACR50781.1	Tetronasin	[29]

Table S6. Summary of cyclase library information regarding the molecular weight in kDA (MW), the extinction coefficient in $m^{-1}*cm^{-1}$(ε), the determined melting temperature in °C (T_M), the obtained protein yields in mg_{Prot} obtained from 12 g of wet cell weight (Protein-Yield), and the peptide mapping results (Protein-MS).

Cyclase	MW	ε	T_M	Protein-Yield	Protein-MS
AbmU	25.645	32430	51	7.9	yes
AbyU	17.742	25440	80.7	4.9	yes
AbsU	19.811	12590	54	2	yes
ChlL	21.412	11460	63.8	5.5	yes
KijU	16.806	14565	64.2	75	yes
LobD1	17.755	10095	54.3	1.3	no
LonU2 (LobU2)	17.588	10095	78.1	1.4	yes
PyrI4	20.546	11585	90	3	yes
QmnH-C	22.784	49055	52.6	0.03	residual
QmnH-N	20.736	27390	66	0.16	residual
TcaU4	19.163	13075	54.4	3.9	yes
Tmn8	20.819	10095	54.5	2	yes
VstJ	16.540	7115	56.4	3	yes
Tsn15	23.512	30940	50.9	25	yes
Cyc01	19.462	34950	47.8	0.2	no
Cyc02	19.471	26720	55.3	0.4	residual
Cyc03	21.634	10095	79.3	24	yes
Cyc04	16.445	29910	44.3	112	yes
mCyc05	23.638	5960	59.2	36	residual
Cyc06	24.207	21805	65.8	0.5	no
mCyc11	15.034	14105	69.6	5.5	yes
Cyc12	39.814	8605	60.6	72	yes
Cyc13	49.534	49640	46.7	13	residual
Cyc15	17.046	29575	75.4	160	yes
Cyc16	19.813	21680	64.3	0.3	no
Cyc17	19.975	16055	-	0	-
CcsF	42.521	56965	62.6	0.2	no
ChlE3	53.185	53065	51.6	22	yes
EupfF	41.585	92360	60.6	0.03	-
gNR600	44.463	77140	64.9	0.2	yes
IccD	33.779	32890	53.3	3.5	residual
ImdH	16.926	17990	64.2	0.7	yes
LepI	44.387	45295	48.1	1.8	yes
LobP3	53.697	50670	54	25	yes
mAsR5	43.355	78505	56.7	0.3	no
MPS	37.311	28420	43.3	12	yes
NgnD	17.619	41940	60.5	243	yes
PhqE	28.593	14690	57.5	0.9	yes
PvhB	41.919	75775	47.8	1.7	yes
PyrE3	49.058	49180	45.9	0.1	no
Sol5	58.147	67395	58.1	0.1	no
SpnF	30.743	45295	58.2	0.4	no
TbtD	41.094	56505	68.4	1.9	yes
TedJ	52.948	56045	55.5	3.5	yes
Tsn11	54.965	60515	45.5	112	yes

Table S7. Summary of X-ray data collection and refinement statistics (pdb code: 8OF7) .

	**Cyc15**
**Data collection**	
Beamline wavelength (Å)	0.9795
Space group	$P4_12_12$
Cell dimensions	
a, b, c (Å)	65.14, 65.14, 180.14
a, b, g, (°)	90.0, 90.0, 90.0
Resolution (Å)	46.10-1.66 (1.69-1.66)[a]
R_{merge}	0.10 (18.07) [a]
No. of reflections	3622415 (180766) [a]
No. of unique reflections	46918 (2267) [a]
$I/\sigma I$	22.7 (0.1) [a]
$CC_{1/2}$	1.0 (0.30) [a]
Completeness (%)	100.00 (99.65) [a]
Redundancy	18.8 (6.6) [a]
**Refinement**	
R_{work}/R_{free}	18.9/21.5
No. of atoms	
Protein	2016
Ligand/ion	102
Water	82
B factors Å^2	
Protein	62.0
Ligand/ion	87.9
Water	67.0
Root mean square deviations	
Bond lengths (Å)	0.012
Bond angles (°)	1.86
Ramachandran favoured (%)	99.2
Ramachandran outliers (%)	0

[a] Values in parentheses are for highest resolution shell

S5 References

[1] a) M. J. Byrne, N. R. Lees, L. C. Han, M. W. van der Kamp, A. J. Mulholland, J. E. Stach, C. L. Willis, P. R. Race, *J. Am. Chem. Soc.* **2016**, *138*, 6095-6098; b) N. S. Berrow, D. Alderton, S. Sainsbury, J. Nettleship, R. Assenberg, N. Rahman, D. I. Stuart, R. J. Owens, *Nucleic Acids Res.* **2007**, *35*, e45.

[2] J. J. Almagro Armenteros, K. D. Tsirigos, C. K. Sonderby, T. N. Petersen, O. Winther, S. Brunak, G. von Heijne, H. Nielsen, *Nat. Biotechnol.* **2019**, *37*, 420-423.

[3] a) J. A. Gerlt, J. T. Bouvier, D. B. Davidson, H. J. Imker, B. Sadkhin, D. R. Slater, K. L. Whalen, *Biochim. Biophys. Acta* **2015**, *1854*, 1019-1037; b) R. Zallot, N. Oberg, J. A. Gerlt, *Biochemistry* **2019**, *58*, 4169-4182.

[4] P. Shannon, A. Markiel, O. Ozier, N. S. Baliga, J. T. Wang, D. Ramage, N. Amin, B. Schwikowski, T. Ideker, *Genome Res.* **2003**, *13*, 2498-2504.

[5] K. Katoh, D. M. Standley, *Mol. Biol. Evol.* **2013**, *30*, 772-780.

[6] B. Q. Minh, H. A. Schmidt, O. Chernomor, D. Schrempf, M. D. Woodhams, A. von Haeseler, R. Lanfear, *Mol. Biol. Evol.* **2020**, *37*, 1530-1534.

[7] H. Venselaar, R. P. Joosten, B. Vroling, C. A. Baakman, M. L. Hekkelman, E. Krieger, G. Vriend, *Eur. Biophys. J.* **2010**, *39*, 551-563.

[8] J. Jumper, R. Evans, A. Pritzel, T. Green, M. Figurnov, O. Ronneberger, K. Tunyasuvunakool, R. Bates, A. Zidek, A. Potapenko, A. Bridgland, C. Meyer, S. A. A. Kohl, A. J. Ballard, A. Cowie, B. Romera-Paredes, S. Nikolov, R. Jain, J. Adler, T. Back, S. Petersen, D. Reiman, E. Clancy, M. Zielinski, M. Steinegger, M. Pacholska, T. Berghammer, S. Bodenstein, D. Silver, O. Vinyals, A. W. Senior, K. Kavukcuoglu, P. Kohli, D. Hassabis, *Nature* **2021**, *596*, 583-589.

[9] F. W. Studier, *Protein Expr. Purif.* **2005**, *41*, 207-234.

[10] J. K. Goodman, C. G. Zampronio, A. M. E. Jones, J. R. Hernandez-Fernaud, *Proteomics* **2018**, *18*, e1800236.

[11] G. Winter, C. M. Lobley, S. M. Prince, *Acta Crystallogr. D Biol. Crystallogr.* **2013**, *69*, 1260-1273.

[12] L. Potterton, J. Agirre, C. Ballard, K. Cowtan, E. Dodson, P. R. Evans, H. T. Jenkins, R. Keegan, E. Krissinel, K. Stevenson, A. Lebedev, S. J. McNicholas, R. A. Nicholls, M. Noble, N. S. Pannu, C. Roth, G. Sheldrick, P. Skubak, J. Turkenburg, V. Uski, F. von Delft, D. Waterman, K. Wilson, M. Winn, M. Wojdyr, *Acta Crystallogr. D Struct. Biol.* **2018**, *74*, 68-84.

[13] A. Vagin, A. Teplyakov, *Acta Crystallogr. D Biol. Crystallogr.* **2010**, *66*, 22-25.

[14] P. Emsley, B. Lohkamp, W. G. Scott, K. Cowtan, *Acta Crystallogr. D Biol. Crystallogr.* **2010**, *66*, 486-501.

[15] G. N. Murshudov, P. Skubak, A. A. Lebedev, N. S. Pannu, R. A. Steiner, R. A. Nicholls, M. D. Winn, F. Long, A. A. Vagin, *Acta Crystallogr. D Biol. Crystallogr.* **2011**, *67*, 355-367.

[16] a) R. A. Friesner, J. L. Banks, R. B. Murphy, T. A. Halgren, J. J. Klicic, D. T. Mainz, M. P. Repasky, E. H. Knoll, M. Shelley, J. K. Perry, D. E. Shaw, P. Francis, P. S. Shenkin, *J. Med. Chem.* **2004**, *47*, 1739-1749; b) T. A. Halgren, R. B. Murphy, R. A. Friesner, H. S. Beard, L. L. Frye, W. T. Pollard, J. L. Banks, *J. Med. Chem.* **2004**, *47*, 1750-1759.

[17] C. O. Marsh, N. R. Lees, L. C. Han, M. J. Byrne, S. Z. Mbatha, L. Maschio, S. Pagden-Ratcliffe, P. W. Duke, J. E. M. Stach, P. Curnow, C. L. Willis, P. R. Race, *ChemCatChem* **2019**, *11*, 5027-5031.

[18] J. Tu, S. Li, J. Chen, Y. Song, S. Fu, J. Ju, Q. Li, *Microb. Cell Fact.* **2018**, *17*.

[19] X. Wang, S. I. Elshahawi, W. Cai, Y. Zhang, L. V. Ponomareva, X. Chen, G. C. Copley, J. C. Hower, C. G. Zhan, S. Parkin, J. Rohr, S. G. Van Lanen, K. A. Shaaban, J. S. Thorson, *J. Nat. Prod.* **2017**, *80*, 1141-1149.

[20] X. Y. Jia, Z. H. Tian, L. Shao, X. D. Qu, Q. F. Zhao, J. Tang, G. L. Tang, W. Liu, *Chem. Biol.* **2006**, *13*, 575-585.

[21] H. Zhang, J. A. White-Phillip, C. E. Melancon, H. J. Kwon, W. L. Yu, H. W. Liu, *J. Am. Chem. Soc.* **2007**, *129*, 14670-14683.

[22] C. Yue, J. Niu, N. Liu, Y. Lü, M. Liu, Y. Li, M. Wang, M. Shao, S. Qian, Y. Bao, Y. Huang, *Pak. J. Pharm. Sci.* **2016**, *29*, 287-293.

[23] S. Li, J. Xiao, Y. Zhu, G. Zhang, C. Yang, H. Zhang, L. Ma, C. Zhang, *Org. Lett.* **2013**, *15*, 1374-1377.

[24] Z. Tian, P. Sun, Y. Yan, Z. Wu, Q. Zheng, S. Zhou, H. Zhang, F. Yu, X. Jia, D. Chen, A. Mándi, T. Kurtán, W. Liu, *Nat. Chem. Biol.* **2015**, *11*, 259-265.

[25] H. Y. He, H. X. Pan, L. F. Wu, B. B. Zhang, H. B. Chai, W. Liu, G. L. Tang, *Chem. Biol.* **2012**, *19*, 1313-1323.

[26] J. Fang, Y. Zhang, L. Huang, X. Jia, Q. Zhang, X. Zhang, G. Tang, W. Liu, *J. Bacteriol.* **2008**, *190*, 6014-6025.

[27] Y. Demydchuk, Y. Sun, H. Hong, J. Staunton, J. B. Spencer, P. F. Leadlay, *ChemBioChem* **2008**, *9*, 1136-1145.

[28] T. Hashimoto, J. Hashimoto, K. Teruya, T. Hirano, K. Shin-ya, H. Ikeda, H. W. Liu, M. Nishiyama, T. Kuzuyama, *J. Am. Chem. Soc.* **2015**, *137*, 572-575.

[29] R. Little, F. C. R. Paiva, R. Jenkins, H. Hong, Y. Sun, Y. Demydchuk, M. Samborskyy, M. Tosin, F. J. Leeper, M. V. B. Dias, P. F. Leadlay, *Nat. Catal.* **2019**, *2*, 1045-1054.

[30] F. Sievers, A. Wilm, D. Dineen, T. J. Gibson, K. Karplus, W. Li, R. Lopez, H. McWilliam, M. Remmert, J. Soding, J. D. Thompson, D. G. Higgins, *Mol. Syst. Biol.* **2011**, *7*, 539.

[31] K. Blin, S. Shaw, A. M. Kloosterman, Z. Charlop-Powers, G. P. van Wezel, M. H. Medema, T. Weber, *Nucleic Acids Res.* **2021**, *49*, W29-W35.

[32] K. Qiao, Y. H. Chooi, Y. Tang, *Metab. Eng.* **2011**, *13*, 723-732.

[33] Q. Chen, J. Gao, C. Jamieson, J. Liu, M. Ohashi, J. Bai, D. Yan, B. Liu, Y. Che, Y. Wang, K. N. Houk, Y. Hu, *J. Am. Chem. Soc.* **2019**, *141*, 14052-14056.

[34] R. C. Jadulco, M. Koch, T. B. Kakule, E. W. Schmidt, A. Orendt, H. He, J. E. Janso, G. T. Carter, E. C. Larson, C. Pond, T. K. Matainaho, L. R. Barrows, *J. Nat. Prod.* **2014**, *77*, 2537-2544.

[35] Z. Zhang, C. S. Jamieson, Y. L. Zhao, D. Li, M. Ohashi, K. N. Houk, Y. Tang, *J. Am. Chem. Soc.* **2019**, *141*, 5659-5663.

[36] I. Drulyte, J. Obajdin, C. H. Trinh, A. P. Kalverda, M. W. van der Kamp, G. R. Hemsworth, A. Berry, *IUCrJ* **2019**, *6*, 1120-1133.

[37] M. Ohashi, F. Liu, Y. Hai, M. Chen, M. C. Tang, Z. Yang, M. Sato, K. Watanabe, K. N. Houk, Y. Tang, *Nature* **2017**, *549*, 502-506.

[38] B. Zhang, K. B. Wang, W. Wang, X. Wang, F. Liu, J. Zhu, J. Shi, L. Y. Li, H. Han, K. Xu, H. Y. Qiao, X. Zhang, R. H. Jiao, K. N. Houk, Y. Liang, R. X. Tan, H. M. Ge, *Nature* **2019**, *568*, 122-126.

[39] R. Schor, C. Schotte, D. Wibberg, J. Kalinowski, R. J. Cox, *Nat. Commun.* **2018**, *9*, 1963.

[40] C. R. Guimaraes, M. Udier-Blagovic, W. L. Jorgensen, *J. Am. Chem. Soc.* **2005**, *127*, 3577-3588.

[41] Y. Ding, S. Gruschow, T. J. Greshock, J. M. Finefield, D. H. Sherman, R. M. Williams, *J. Nat. Prod.* **2008**, *71*, 1574-1578.

[42] D. Tan, C. S. Jamieson, M. Ohashi, M. C. Tang, K. N. Houk, Y. Tang, *J. Am. Chem. Soc.* **2019**, *141*, 769-773.

[43] K. Kasahara, T. Miyamoto, T. Fujimoto, H. Oguri, T. Tokiwano, H. Oikawa, Y. Ebizuka, I. Fujii, *ChemBioChem* **2010**, *11*, 1245-1252.

[44] H. J. Kim, M. W. Ruszczycky, S. H. Choi, Y. N. Liu, H. W. Liu, *Nature* **2011**, *473*, 109-112.

[45] D. P. Cogan, G. A. Hudson, Z. Zhang, T. V. Pogorelov, W. A. van der Donk, D. A. Mitchell, S. K. Nair, *Proc. Natl. Acad. Sci. USA* **2017**, *114*, 12928-12933.

[46] T. Gverzdys, G. Kramer, J. R. Nodwell, *Bioorg. Med. Chem.* **2016**, *24*, 6269-6275.
